# Distinct descending and biomechanical influences on interlimb coordination in mice

**DOI:** 10.1016/j.isci.2026.115008

**Published:** 2026-02-16

**Authors:** Zane Mitrevica, Andrew J. Murray

**Affiliations:** 1 Sainsbury Wellcome Centre for Neural Circuits and Behaviour, University College London, London W1T 4JG, UK

**Keywords:** biomechanics, Neuroscience

## Abstract

Interlimb coordination, or gait, is a hallmark of locomotion, but has been challenging to study due to its partial dependence on speed and the difficulty of reliably evoking the full gait spectrum in genetically amenable quadrupeds such as mice. To address this, we developed a head-fixed locomotor paradigm that decouples the speed- and leg loading-related effects on gait by combining optogenetic stimulation in the cuneiform nucleus with head height and surface slope modulation. This approach revealed a largely speed-independent shift in homolateral phase preference from strict alternation to a quarter-of-phase more synchronized coordination upon a rearward redistribution of load. This load-related effect was observed regardless of hindlimb phase and aligned with changes in limb support patterns. These findings highlight how quadrupeds use biomechanical input to coordinate limbs across speeds and environments, and serve as an entry point to a behaviour-driven study of gait circuits.

## Highlights

• Speed- and leg load-related effects on interlimb coordination are explored in mice
• Hind-heavy loading correlates with up to a quarter-phase homolateral synchronization
• The effect is largely speed-independent and aligns with changes in support patterns

## Graphical abstract

**Figure:**
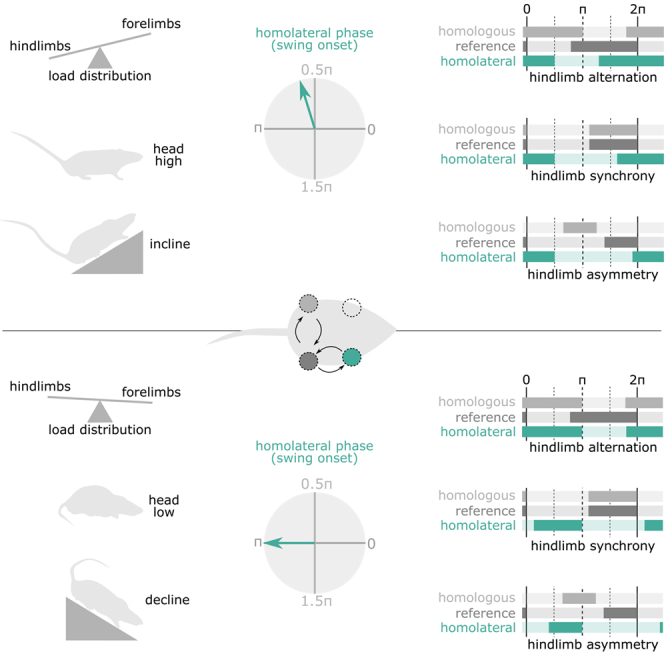

## Introduction

Locomotion is one of the principal motor outputs of the mammalian nervous system, essential for behaviors as diverse as foraging, migration, and escape. Successful execution of all these tasks relies on the continual adjustment of locomotor gait in line with the behavioral demand for speed, as well as the terrain. Failure in this process would disrupt locomotor smoothness, raise its energetic cost, and increase the risk of injury due to skeletal stress.^1^^,^^2^ Quadrupeds avoid these scenarios, in part, by switching from left-right alternating gaits, such as walk and trot, to gaits with partial or full bilateral synchrony, such as gallop and bound, as they increase movement speed.^3^^,^^4^^,^^5^^,^^6^ However, the relationship between speed and gait is not deterministic,^6^^,^^7^ and gait transitions have also been associated with several other factors, including locomotor energetics,^8^^,^^9^ gait viability,^10^ and biomechanical variables.^11^^,^^12^^,^^13^ For example, experiments with sloped surfaces or load-bearing dogs have shown that load placement along the anteroposterior body axis affects homolateral coordination,^14^^,^^15^^,^^16^ a result corroborated by simulations and robotics.^17^^,^^18^ In a separate line of work with load-bearing horses, an increase in total load has been linked to a lowering of the trot-to-gallop transition speed,^11^ suggesting a broader biomechanical effect on gait selection. Indeed, the exact trigger of gait transitions—whether total load, its distribution, or another factor—remains unclear, as does the full scope of biomechanical influences. This gap persists partly because causal perturbations, such as those enabled by transgenic techniques, are not feasible in the large mammals typically suitable for biomechanical manipulations.

At the same time, a lot of the recent excitement in locomotor research comes from the application of transgenic techniques in mice, with biomechanical considerations taking a backseat. The typical experimental pipeline begins with a genetically or spatially restricted neural perturbation and relies on standard locomotor paradigms to assess the role of the affected neurons. While many features of locomotion, including initiation, reorientation, and speed modulation, have thus been mapped onto well-defined neuron types in the brainstem and the spinal cord,^19^^,^^20^^,^^21^^,^^22^^,^^23^^,^^24^ the effects on gait are rarely so striking that they can be dissociated from speed-related changes.^6^^,^^25^ More often, it is not clear whether an experimental effect on interlimb coordination reflects a primary effect on gait or a secondary effect of a speed disturbance.^26^^,^^27^^,^^28^^,^^29^ Progress toward understanding the computational logic and neural implementation of quadrupedal gait, therefore, requires that the neural approaches available in mice are integrated with speed-independent, biomechanical manipulations. Moreover, a thorough investigation of gait in mice requires consistent sampling of stepping patterns across the full gait range characteristic of this species. However, in mice, standard locomotor paradigms such as motorized treadmill and self-initiated overground locomotion do not reliably elicit the high speeds typically associated with the frequent expression of left-right synchronized limb coordination.^7^^,^^28^

Here, we address these challenges by developing a head-fixed locomotor paradigm that decouples the speed- and leg loading-related effects on interlimb phase through head height and surface slope modulation, and reliably evokes the full spectrum of mouse gait through unilateral optogenetic stimulation in the cuneiform nucleus (CnF). This neural perturbation has been shown to induce locomotion at a wide range of speeds and gaits, depending on stimulation frequency,^19^^,^^21^ so we leverage it to systematically diversify locomotor output. Our paradigm reveals a largely speed-independent shift in homolateral limb coordination from strict alternation to a quarter-of-phase more synchronized pattern at upward body orientations and uphill slopes. This shift correlates with a posteriorward displacement of the animal’s center of support (CoS), quantified as the load-weighted average position of limb contact points using vertical ground reaction force measurements under stationary conditions. The effect is also robust across left-right coordination patterns and different from the load-related influences on hindlimb phase (see supplemental information). Our findings demonstrate distinct top-down and biomechanical influences on interlimb coordination and raise intriguing questions for future investigation of the underlying neural circuits.

## Results

### A combination of biomechanical and optogenetic modulation enables the systematic study of interlimb coordination

**Figure 1:**
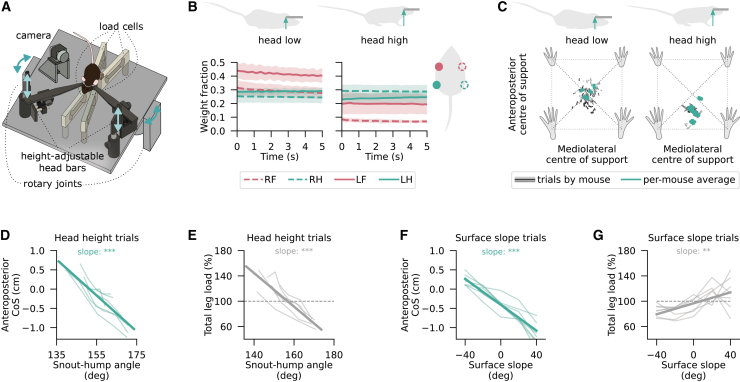
Head height and surface slope manipulation effectively modulate total load and its anteroposterior distribution (A) Schematic representation of the leg load measurement setup that allows the modulation of mouse head height (light arrows) and surface slope (dark arrows). (B) Average fraction of body weight placed on each of the four limbs by a representative mouse during 5 s standstills at two head heights 15 mm apart. Shaded areas show 95% confidence intervals across trials. (C) Center of support along the anteroposterior and mediolateral body axes at a low (*left*) and high (*right*) head position. Gray traces show individual trials with shade indicative of mouse identity (*n* = 7 mice). Within-mouse means are shown in teal. (D and E) Anteroposterior center of support (D; *mean slope*±*SEM: -0.027*±*0.001 cm/deg, t test with Satterthwaite correction t(7)=-15.8, p=1*×*10*^*−6*^) and total leg load (E; *slope: -2.6*±*0.2%/deg, t(8)=-13.5, p=1*×*10*^*−6*^) as a function of snout-hump angle. Zero CoS reflects equal weight placed on forelimbs and hindlimbs. Shown are trial averages of individual mice (thin lines), along with a linear mixed-effects regression line (thick lines). (F–G) Same as (D and E) but showing anteroposterior CoS (F; *slope: -0.0096*±*0.0007 cm/deg, t(6)=-13.8, p=5*×*10*^*−6*^) and total leg load (G; *slope: 0.43*±*0.09%/deg, t(6)=4.6, p=0.004*) as a function of surface slope in the slope trials. Statistical significance refers to t-tests with thresholds: ∗ p<0.05, ∗∗ p<0.01, ∗∗∗ p<0.001. See also Figure S1.

A behaviour-driven investigation of the gait control system requires an ability to decouple gait from speed and reliably evoke the full spectrum of interlimb coordination patterns relevant to the species under study. Theoretical and empirical evidence from quadrupeds of distinct body builds suggests that the interlimb coordination of an animal is influenced by the absolute or relative load borne by its legs.^9^^,^^10^^,^^11^^,^^18^^,^^30^^,^^31^ Building on these insights and leveraging the head fixation techniques available in mice, we sought to design a locomotor paradigm that enables the modulation of the anteroposterior leg load distribution of these animals by altering their head height. Specifically, we expected lower head positions to increase the fraction of body weight borne by the forelimbs, and vice versa. To verify this hypothesis, we used four single-point load cells to measure the vertical ground reaction force borne by each leg of stationary head-fixed mice as their head height was changed over a 25 mm range (Figures 1A and 1B). As expected, the fraction of weight borne by the forelimbs increased by 69±8% when the head was positioned closer to the ground compared to the maximum tested head height (Figure S1A). The change in hindlimb load with increasing head height was small in comparison (6±4%) and not statistically significant (Figure S1B). Instead, the total detected load decreased as a function of head height, indicating that mice primarily transferred their body weight between the forelimbs and the head fixation apparatus (Figure S1C). This interaction with the experimental setup represented a clear departure from the animals’ natural, unrestrained locomotor state. Still, as required by the new locomotor paradigm, it led to a redistribution of load across the legs, which we quantified as the displacement of CoS. As the head was raised to higher positions, CoS shifted primarily along the anteroposterior body axis, toward the hindlimbs (Figure 1C). Specifically, a 25 mm rise in head position displaced CoS posteriorly by 1.1±0.2 cm on average (*p* = 0.02, *t test*), and this relationship was well-approximated by an exponential decay function (Figure S1D). No significant change was observed along the mediolateral axis (Figure S1E). These results confirm that manipulating the head height of head-fixed mice is an effective way of modulating forelimb load, total load, and its anteroposterior distribution.

Despite head fixation, mice were still able to modulate their body weight distribution through postural adjustments not captured by head height alone. To account for these postural variations, which we assumed were largely reflected in spinal curvature, we computed the snout-hump angle, namely the obtuse angle formed by the vector from the hump of the back to the fixed snout (“hump-snout vector”) and a line through the snout coordinate parallel to the treadmill surface. Similar to the load-related variables considered before, snout-hump angle varied curvilinearly with head height, approaching an asymptote of 180° at the highest tested head positions (Figure S1F). Importantly, the relationships between snout-hump angle and both anteroposterior CoS and total leg load were linear, such that these metrics shifted by 0.5±0.2 mm and −2.8±0.2% per degree of snout-hump angle, respectively (Figures 1D and 1E). Taking advantage of these linear associations, we use the snout-hump angle as a proxy for these load-related factors in subsequent analyses of locomotion.

Head height-related variation in both total leg load and the anteroposterior CoS would make it challenging to distinguish whether any locomotor effect that correlates with snout-hump angle was due to changes in the absolute leg load or its distribution. To better discriminate between these mechanical factors, we further sought to modulate leg load distribution by changing the slope of the limb support surface while keeping the head at a fixed medium height. Based on previous work with non-restrained mammals,^13^^,^^16^^,^^32^ we expected that inclines would shift CoS posteriorly by redistributing load from forelimbs to hindlimbs. Indeed, every 10° increase in slope shifted CoS posteriorly by 1.7±0.9 mm, resulting in a total displacement of 1.4±0.1 cm over the tested slope range (Figure 1F), without a significant change along the mediolateral axis (*p* = 0.13). Although our recording of only the vertical force component made the detected fraction of body weight dependent on surface slope, we could still observe an increase in the fraction of weight borne by the hindlimbs (from 0.37±0.02 to 0.92±0.07) and a decrease in the forelimb load fraction (from 0.49±0.02 to 0.21±0.04) as the slope changed from a −40° decline to a 40° incline (Figures S1G and S1H). After adjusting for slope-dependent changes in detectable load, the total load was also found to vary with surface slope, such that mice transferred up to 15±3% of their weight onto the head fixation apparatus on declines (Figure 1G). Importantly, an increase in total detected load was associated with a posteriorward CoS shift in the head height trials and an anteriorward CoS shift in slope trials. Thus, combining both types of load manipulation provides a unique opportunity to distinguish the effects of total load and its distribution, which will be critical in the locomotor experiments described later.

**Figure 2:**
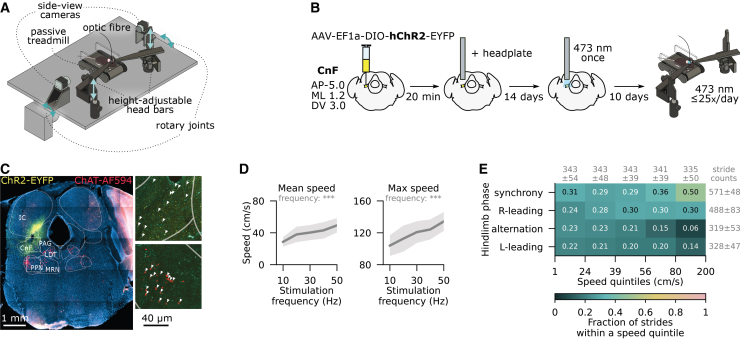
Optogenetic CnF stimulation reliably triggers diverse interlimb coordination patterns (A) Schematic representation of the passive treadmill setup that allows optogenetic CnF stimulation during head height (light arrows) and surface slope (dark arrows) modulation. (B) Experimental strategy and timeline. (C) An example coronal section at the virus injection and optic fiber implantation site, visualized by epifluorescence (*left*, scale bars, 1mm) and confocal (*right: two insets*, scale bars, 40μm) microscopy. The images show enhanced yellow fluorescent protein (YFP) labeling in the opsin-expressing cells (yellow), choline acetyltransferase (ChAT) antibody staining (red), and DAPI staining (blue). CnF, cuneiform nucleus; PPN, pedunculopontine nucleus; MRN, mesencephalic reticular nucleus; PAG, periaqueductal gray; IC, inferior colliculus; LDT, laterodorsal tegmental nucleus. (D) Median (*left; mean slope*±*SEM: 0.45*±*0.06, t test with Satterthwaite correction t(24)=7.9, p=3*×*10*^*−8*^) and maximum (*right; slope: 0.72*±*0.09, t(37)=8.1, p=1*×*10*^*−9*^) speed of optogenetically evoked head-fixed locomotion as a function of optical stimulation frequency, averaged across a range of head fixation heights (*n* = 20 mice). Shown are means with 95% confidence intervals, with statistics referring to the slope coefficient in a linear regression. (E) Fraction of strides with synchronous, alternating (anti-phase), and left- or right-leading hindlimb coordination patterns across speed quintiles on the passive treadmill. Fractions are averaged across mice and normalized within each speed quintile. Marginal stride counts, averaged across mice, are shown along rows and columns. Statistical significance refers to t-tests with thresholds: ∗ p<0.05, ∗∗ p<0.01, ∗∗∗ p<0.001. See also Figure S2.

To study locomotion under specific biomechanical conditions, we replaced the cube-shaped force sensors with a custom-built passive treadmill such that the setup still enabled the independent adjustment of animal head height and surface slope (Figure 2A, Videos S1, S2, S3, and S4). Prior to these locomotor experiments, we expressed channelrhodopsin in the glutamatergic neurons of the cuneiform nucleus (CnF), which have been shown to trigger forward movement at a speed that scales with stimulation frequency^19^^,^^20^^,^^21^ (Figures 2B and 2C, S2A–S2C). Indeed, CnF stimulation at 10–50 Hz reliably evoked locomotion at short latencies (<150 ms in 88% trials) and a running speed that rose linearly by an average of 4.6±0.5 cm/s with every 10 Hz increase in stimulation frequency (Figure 2D). Importantly, this approach consistently elicited diverse interlimb coordination patterns, including 15–23% of strides with hindlimb alternation and 29–36% of strides with left-right synchronization at speeds below <80 cm/s. Only at higher speeds, the prevalence of synchronized hindlimb movement reached 50%, accompanied by sizable proportions of right-leading (30±6%) and left-leading (14±2%) out-of-phase movement (Figure 2E). This diversity of interlimb phases made our paradigm better suited for studying quadrupedal gait than the widely used motorized treadmill, where left-right alternation comprised 59–79% of strides in all speed bands (Figures S2D and S2E). In fact, the range of observed interlimb coordination patterns was, on average, comparable with freely moving escape behavior (Figures S2F–S2I). However, the use of an escape paradigm for the study of gait would be impractical due to sensitization to the stimulus and reduced likelihood of exiting the shelter after a few stimulus exposures. While mice performed 5±1 escapes during the first 1-h session in the open field, the probabilities of escape and shelter departure in the second experimental session dropped by 35% and 83%, respectively, highlighting the challenges of sensitization and reduced engagement over time. Overall, these observations illustrate how the ability to consistently elicit diverse interlimb coordination patterns while allowing for biomechanical modulation makes optogenetically induced locomotion in a head-fixed setting better suited for the study of gait than traditional and less constrained locomotor paradigms.

Despite these benefits, unilateral optogenetic stimulation introduced a lateral bias in hindlimb and forelimb coordination. When homologous coordination was neither strictly alternating nor synchronous, unilateral CnF activation, on average, promoted a stepping sequence in which the ipsilateral limb touched down more anteriorly and delivered the final propulsive push (Figure S2J). Supporting this observation, a binary classifier trained to predict the side of optogenetic stimulation from limb phase data achieved accuracies of 67.7±0.5% and 62.7±2.0% on hindlimb and forelimb phase data, respectively (Figure S2K). No such bias was observed for homolateral phase data (classifier accuracy: 50.8±1.1%, *p* = 0.16), indicating no systematic lateralization along this axis. Since minor asymmetries were found during non-stimulated motorized treadmill locomotion of mice that had undergone the same surgical procedures (Figures S2L and S2M; classifier accuracies: 52–54%), subtle biases in left-right coordination may arise from unilateral CnF tissue damage alone. Still, the much stronger phase bias observed during optogenetically stimulated locomotion was likely attributable to asymmetric descending activation. These biases do not diminish the potential utility of the passive treadmill paradigm but should be corrected for through data mirroring or stratification in subsequent analyses of locomotion.

### Changes in posture and surface slope influence homolateral coordination, with limited effects of speed

**Figure 3:**
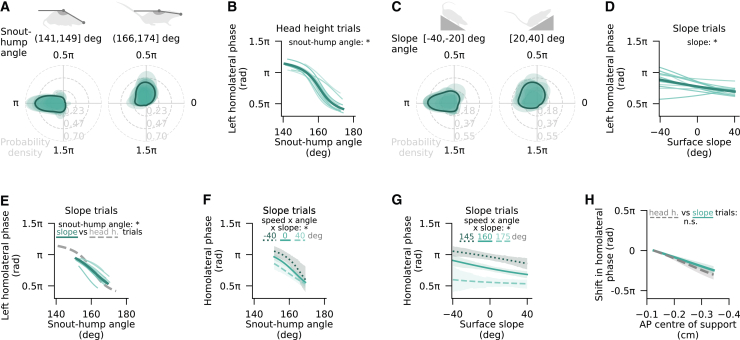
Changes in posture and surface slope influence homolateral coordination, with limited effects of speed (A and C) Von Mises-smoothed distributions of left forelimb (LF) phase relative to left hindlimb (LH) in two intervals of snout-hump angles (A) and surface slopes (C), shown for individual mice (shaded regions; *n* = 12 mice) and as averages across mice (solid outlines). Limb phase values of 0 and π rad reflect limb synchrony and alternation, respectively. Data from mice with fewer than 40 strides per category were excluded from the respective plot. (B and D) Left homolateral phase as a function of snout-hump angle (B; $HPD_{\mathrm{SSDO}}$=(0.96,1.15)) and surface slope (D; $HPD_{\mathrm{SSDO}}$=(1.04,1.31)) with LH as the reference limb at median speed. Shown are circular-linear mixed-effects regression fits to individual mouse data (random effects; light traces) and the average (fixed effect; dark trace). (E) Left homolateral phase as a function of snout-hump angle in surface slope trials, with LH as the reference limb at median speed ($HPD_{\mathrm{SSDO}}$=(0.98,1.23)). Shown are circular-linear mixed-effects regression fits to single mouse data (random effects; light traces) and the average (fixed effect; dark trace). The analogous fixed effect of snout-hump angle in head height trials is shown by the dashed gray trace for reference. (F and G) Homolateral phase as a function of snout-hump angle (F) and surface slope (G) in slope trials at median speed, showing the interaction effect between speed, slope, and snout-hump angle determined through circular-linear mixed-effects regression ($HPD_{\mathrm{SSDO}}$=(-1.20,-0.99)). (H) Homolateral phase shift as a function of the anteroposterior CoS position in surface slope (teal) and head height (gray) trials, analyzed over the range of CoS positions observed in all mice in response to changes in either slope or snout-hump angle (*p* = 0.1). This range was constrained by the snout-hump angles seen in slope trials. Statistical significance criterion (Bayesian posterior interval): ∗ $HPD_{\mathrm{SSDO}}$ interval does not include zero, n.s. otherwise. See also Figures S3–S6.

Previous research in living and simulated quadrupeds has linked load redistribution along the anteroposterior body axis to a shift in homolateral coordination, although the nature of this shift has differed across studies.^14^^,^^15^^,^^16^^,^^17^^,^^18^ Having established the snout-hump angle as a mouse size-invariant proxy for leg load and its anteroposterior distribution, we explored the relationship of this variable and homolateral limb phase. Over a two-week period, mice (*n* = 20, at least 1000 strides each) were optogenetically induced to locomote on the passive treadmill while their head was fixed at different heights over a 25 mm range. A broad range of interlimb coordination patterns was observed across experimental conditions (Figure S3). Across locomotor speeds, there was a notable prevalence of anti-phase homolateral coordination at hunched postures (mean±sd: 1.06±0.11 π rad) and a quarter-of-phase more synchronized coordination at upward oriented snout-hump orientations (0.54±0.13 π rad; Figure 3A), with the majority of animals (*n* = 12) displaying a unimodal phase distribution at both extremes of the snout-hump angle range (Figure S4A).

To dissociate the relative contributions of snout-hump angle and speed to homolateral coordination, we fit a circular-linear regression model of left homolateral phase as a function of the aforementioned variables, using random effects to capture individual variation across mice. This analysis supported snout-hump angle as a strong predictor of left homolateral limb phase, such that an upward shift in the hump-snout vector was associated with an approximately inverse sigmoidal phase transition pattern when visualized in linear coordinates (Figure 3B). This effect on left homolateral phase preference was highly conserved across mice and qualitatively preserved regardless of the hindlimb coordination pattern, although slightly (0.1 π rad) more pronounced during left-right synchronized and asymmetric hindlimb stepping (Figures S4B and S4C). Hindlimb coordination itself showed only indirect, and comparatively weak, dependence on biomechanical variables, specifically total load and a posture-related factor (see Figure S5 and the accompanying text in supplemental information), but to account for its significant influence on homolateral phase, all subsequent analyses either explicitly included hindlimb phase as a predictor or were based on subsampled datasets balanced for strides with alternating (1±0.2 π rad), synchronized (0±0.2 π rad), and asymmetric (all other phases) hindlimb coordination. The asymmetric subset included both left- and right-leading hindlimb phases as the leading limb had no significant effect on homolateral coordination (see Figure S4B). In addition, since the average effect of snout-hump angle on homolateral phase was independent of the side of the reference limb (Figure S4D), further analyses were performed on data pooled across right and left homolateral phases.

In contrast to snout-hump angle, the effects of speed and speed-angle interaction, while statistically significant, amounted to a homolateral phase change of less than 0.1 π rad across the observed speed range, with higher speeds corresponding to a slight delay in the homolateral forelimb step cycle (Figure S4E). In fact, the significant speed-angle interaction likely reflects the speed-dependence of hindlimb phase, considering that, within a phase category, the effect of snout-hump angle is statistically independent of speed ($HPD_{\mathrm{SSDO}}$=(-0.82,0.64)). These findings suggest the presence of a load-driven gait modulation mechanism that functions largely separately from the well-established speed control pathway originating from the midbrain. However, given that the snout-hump angle in a head-fixed context is linked to changes in both absolute and relative leg load, the current results alone cannot determine whether the partial synchronization of homolateral movements at larger snout-hump angles stems from a reduction in total load, its posteriorward redistribution, or load-independent upward arching of the back.

To distinguish between these possibilities, the same mice performed trials on surfaces with varying slopes while their head remained fixed at a constant height. If the homolateral limb phase was primarily influenced by total leg load, declines would be expected to promote greater homolateral synchronized due to partial weight transfer onto the head fixation apparatus, mirroring the effect of upward-oriented hump-snout vectors in head height trials. Alternatively, if the key factor was the anteroposterior load distribution, declines would likely correlate with an increased homolateral phase lag, resembling the effect of hunched postures in head height trials. Finally, if the previously observed effects of snout-hump angle on limb phase were solely attributable to posture, homolateral coordination would remain unaffected by a change in surface slope.

In line with leg load distribution being a relevant factor in homolateral coordination, slope trials revealed a shift in the left homolateral phase preference from nearly anti-phase coordination on steep declines (0.96±0.14 π rad) to more synchronized movement on steep inclines (0.64±0.17 π rad; Figures 3C and S4F). Circular-linear regression analysis with surface slope as an additional predictor supported this variable as a significant determinant of homolateral phase regardless of the reference limb or hindlimb phase (Figures 3D and S4G–S4I), while the influence of speed remained relatively minor (Figure S4J). Although the interaction of speed and slope was statistically significant, it contributed a minimal phase difference (∼0.01 π rad) across an 80° change in slope, suggesting a negligible practical relevance. Conversely, despite the head being fixed at a single height, the snout-hump angle had a notable influence on homolateral coordination. Phase shifted by 0.45±0.05 π rad toward more synchronized homolateral motion at upward orientations of the hump-snout vector, reflecting a qualitatively similar, but smaller, effect compared to head height trials (Figure 3E). This difference in effect attributed to snout-hump angle in head height and slope trials would be expected to be entirely captured by the effect of slope if load distribution was the key variable influencing the homolateral phase. Indeed, joint regression analysis on both trial types showed no significant effect of trial type (Figure S4K), supporting the hypothesis of load distribution-dependent homolateral coordination.

There are three further arguments that strengthen this hypothesis. First, the effects of snout-hump angle and slope were interdependent, with each variable exerting a stronger influence when the other contributed to increased forelimb loading (Figures 3F and 3G). This interaction suggests that both perturbations may affect homolateral coordination through a shared mechanism, such as anteroposterior load distribution. Second, incorporating body weight as a predictor in the regression analysis indicated a tendency for the homolateral forelimb phase to be delayed by larger weight (Figure S4L). Given that heavier mice need a greater redistribution of body mass to shift the CoS over a particular distance, this observation aligns with the idea of load distribution being a key modulator of the homolateral phase. Finally, having both load sensor and locomotor data for each trial type enabled the translation of changes in snout-hump angle and slope into shifts in anteroposterior CoS. This, in turn, allowed for analysis of whether the relationship between homolateral coordination and CoS was consistent across biomechanical manipulations. A posteriorward ∼0.4 cm shift in the CoS was associated with a quarter-of-phase more synchronized homolateral coordination irrespective of the manipulation method (*p* = 0.1; Figure 3H), supporting the hypothesis that anteroposterior leg load distribution influences homolateral limb phase. Altogether, these results suggest that body biomechanics have a speed-independent handle over homolateral limb coordination.

A key limitation of CoS-based equivalation of homolateral phase data is its reliance on snout-hump angle and surface slope having the same relationship to CoS on the passive treadmill and the force sensors. Since direct load recording on the treadmill was not technically feasible, we used postural factors as surrogate measures to compare biomechanical variables across setups. Notably, the head height-dependence of snout-hump angle was consistent between the force sensors and the passive treadmill (Figure S6A), suggesting that a given snout-hump angle reflects a similar anteroposterior CoS and leg load in both conditions. In contrast, slope modulation elicited distinct postural adaptations in the two setups. On the force sensors, mice compensated for the incline-related posteriorward shift in CoS by positioning their feet more anteriorly (Figure S6B) and adopting a more hunched posture (Figure S6C), which likely enhanced their stability on the small surface area of the load cells. On the treadmill, inclines were associated with a posteriorward shift in foot placement and a slightly more upright posture. Given that these adaptations support uphill movement, the differing responses to slope modulation plausibly stem from the mobile nature of the treadmill surface and the potential for movement it provides. Consequently, force sensor-derived biomechanical measurements may not directly translate to the treadmill, necessitating caution in cross-setup comparisons. Nevertheless, the head height trials support the hypothesis that homolateral coordination is influenced by a load-related variable in a manner largely independent of locomotor speed.

### Homolateral limb coordination is linked to changes in limb support patterns

The observed relationship between anteroposterior load distribution and homolateral limb coordination raises questions about the mechanism linking these variables and their potential benefits to the animal. For instance, previous research has attributed homolateral synchronization to increased stride length, suggesting that it serves to prevent limb collision.^33^ Alternatively, the effect of load distribution on homolateral phase could be mediated by changes in the proportion of stride spent in stance, also known as the duty factor. Specifically, as shown in rats and cats,^34^^,^^35^^,^^36^ mice might increase the duty factor of more heavily loaded legs to distribute muscle forces over a longer period, mitigating the peak forces experienced by a leg and thereby reducing the risk of strain-related injury. In the present experiments, both stride length and the duty factor ratio between hindlimbs and forelimbs showed statistically significant effects on the left homolateral phase, as well as significant interaction effects with snout-hump angle, surface slope, or both (Figures S7A–S7F). However, the overall effect of these variables amounted to just 0.1π rad or less over the ranges of observed snout-hump angles and surface slopes. These findings suggest that stride length and duty factor ratio may contribute to the observed relationship between load distribution and homolateral phase, but are likely not its primary mediators.

**Figure 4:**
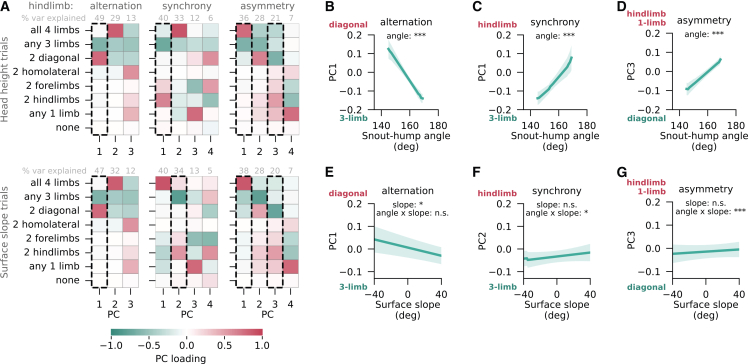
Homolateral limb coordination is linked to changes in limb support patterns (A) Loadings of the first 3–4 principal components collectively representing at least 90% of the variance in limb support patterns during alternating (*left*), synchronized (*middle*), or asymmetric (*right*) hindlimb stepping in head height (*top*) and surface slope (*bottom*) trials on the passive treadmill. The variance explained by each component is shown above the heatmap. Rectangles with black dashed boundaries mark the PCs that were significantly predicted by snout-hump angle, surface slope, or their interaction in a linear mixed-effects regression, and that showed a qualitatively consistent effect across trial types. (B–D) Limb support data from head height trials projected onto PC1 of strides with hindlimb alternation (B; *snout-hump angle effect: t=-7.4, p=1*×*10*^*−5*^), PC1 of strides with hindlimb synchrony (C; *t=5.2, p=2*×*10*^*−4*^), and PC3 of strides with hindlimb asymmetry (D; *snout-hump angle effect: t=9.3, p=5*×*10*^*−6*^), plotted as a function of snout-hump angle. These PCs correspond to the marked PCs in (A). Shown are means across mice, with 95% confidence intervals. (E–G) Same as (B-D) but using data from surface slopes trials. Separately shown are the principal component axes identified in strides with alternating (E; *slope effect: t=-2.5, p=0.03; angle-slope interaction effect: t=1.8, p=0.07*), synchronized (F; *slope: t=1.0, p=0.3; interaction: t=2.5, p=0.01*), and asymmetric (G; *slope: t=1.0, p=0.4; interaction: t=4.7, p=3*×*10*^*−6*^) hindlimb stepping. All analyses refer to the same animals as Figure 3 (*n* = 12 mice). Statistical significance criterion (Bayesian posterior interval): ∗ $HPD_{\mathrm{SSDO}}$ interval does not include zero, n.s. otherwise. See also Figure S7.

The relationship between anteroposterior CoS and homolateral phase may itself serve as an injury prevention strategy. By engaging the homolateral forelimb earlier in the step cycle, mice could either distribute load across more limbs or transition from a diagonally synchronized trotting gait to a footfall pattern that optimizes energy efficiency through distributed contacts.^37^ To explore this hypothesis, we analyzed the primary axes of limb support variance during left-right alternating, synchronized, or asymmetric locomotion on the passive treadmill and examined their relationship with snout-hump angle and surface slope. Over 90% of the variance was explained by the first three or four principal components (PCs) for all left-right coordination patterns (Figure 4A). For an axis of support pattern variance to be plausibly driven by load-related changes in the homolateral phase, it should exhibit a congruent and statistically significant relationship with CoS-related variables across head height and slope trials. During hindlimb alternation, the only PC meeting this criterion represented a shift from diagonal to three-limb support at upward snout-hump orientations and slopes (Figures 4B and 4E). Conversely, when the hindlimbs were synchronized, the same biomechanical conditions were associated with greater homologous limb support at the expense of three-limb support (Figures 4C and 4F). Finally, during left-right asymmetric stepping, the PCs that significantly correlated with load-related variables captured exchanges between three- and four-limb support (PC1; *snout-hump angle in head height trials: t=5.7, p=0.0004; slope-angle interaction in slope trials: t=2.7, p=0.007*) and between diagonal and hindlimb- or single-limb support (PC3; Figures 4D and 4G). The former axis is likely driven, at least in part, by head-fixation and a highly imbalanced load distribution, as it is also observed during left-right alternating (PC2) and synchronized (PC2 in head height, PC1 in slope trials) stepping on the passive treadmill, but occurs far less frequently during non-restrained locomotion on the motorized treadmill (Figure S8A). In contrast, the latter axis may demonstrate a transition toward distributed limb support, consistent with the hypothesized role of earlier homolateral limb engagement. For completeness, we also verified that the changes in limb support represented by these PCs were significantly and coherently related to variations in homolateral phase, mirroring its relationships with the examined biomechanical variables (Figures S7G–S7I). At the same time, these PCs explain only 20–49% of the total variance in support data, indicating that a substantial fraction of limb support variability remains incompatible with modulation by anteroposterior load distribution. Collectively, these findings support the hypothesis that load-related changes in homolateral phase occur in concert with shifts in limb support and may serve as a strategy to reduce mechanical strain and energy costs, although other factors also contribute.

### Homolateral coordination during non-restrained locomotion is broadly consistent with modulation by load distribution

**Figure 5:**
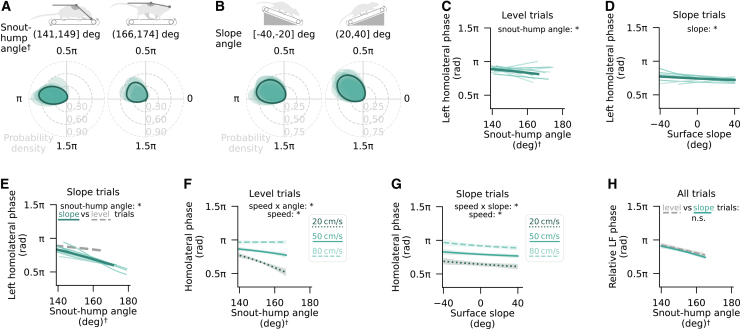
Homolateral coordination during non-restrained locomotion is broadly consistent with modulation by load distribution (A and B) Von Mises-smoothed distributions of LF phase relative to LH in two intervals of snout-hump angles (A, *n* = 12 mice) and surface slopes (B, *n* = 10 mice) recorded on the motorized treadmill. Shown are data for individual mice (shaded regions) and their averages (solid outlines). Phase values of 0 and π rad reflect limb synchrony and alternation, respectively. Data from mice with fewer than 40 strides per category were excluded. (C–E) Left homolateral phase as a function of snout-hump angle (C, E; $HPD_{\mathrm{SSDO}}$=(2.60,2.90)) and surface slope (D; $HPD_{\mathrm{SSDO}}$=(2.30,2.59)) with LH as the reference limb at median speed, using data from level (C) and slope (D, E) trials. Shown are circular-linear mixed-effects regression fits to single mouse data (random effects; light traces) and the average (fixed effect; dark trace). In (E), the mean effect from level trials is also shown (*gray, dashed*). (F and G) Homolateral phase as a function of snout-hump angle (F; $HPD_{\mathrm{SSDO}}$=(2.56,2.81)) and surface slope (G; $HPD_{\mathrm{SSDO}}$=(2.26,2.57)) at three representative speeds, using data from level (F) and slope (G) trials. Shown are the fixed effects from circular-linear mixed-effects regression analysis. Shaded regions represent 95% highest posterior density intervals. Speed-angle interaction $HPD_{\mathrm{SSDO}}$=(-2.83,-2.57), speed-slope interaction $HPD_{\mathrm{SSDO}}$=(-2.55,-2.27). (H) Same as C-E, but using data from both level (*gray, dashed*) and slope (*teal, solid*) trials. Shown are the fixed effects from circular-linear mixed-effects regression analysis with trial type as a predictor ($HPD_{\mathrm{SSDO}}$=(-0.02,0.17)). † indicates that snout-hump angle is measured in non-restrained conditions and is not necessarily directly comparable to the snout-hump angle from head-fixed experiments Statistical significance criterion (Bayesian posterior interval): ∗ $HPD_{\mathrm{SSDO}}$ interval does not include zero, n.s. otherwise. See also Figure S8.

So far, we have shown a significant shift in homolateral coordination in response to changes in snout-hump angle or surface slope during optogenetically evoked, head-fixed locomotion. To assess whether these relationships hold during self-initiated, non-restrained movement, we analyzed locomotion on a level (*n* = 12 mice) and sloped (*n* = 10 mice) motorized treadmill without head-fixation or optogenetic stimulation, with each mouse performing at least 1000 strides. Similar to our observations on the passive treadmill, an upward reorientation of the hump-snout vector correlated to a homolateral phase shift toward synchronization, with phase changing from 0.92±0.07 π rad to 0.79±0.11 π rad as the snout-hump angle increased by 25° (Figure 5A). Likewise, an 80° shift in surface slope resulted in a homolateral phase change from 0.85±0.08 π rad on steep declines to 0.78±0.06 π rad on steep inclines (Figure 5B).

To quantify the relationships between these variables, along with the effects of speed and hindlimb phase, we used circular-linear mixed-effects regression with left homolateral phase as the outcome variable, as before. This analysis confirmed that more upward-oriented snout-hump angles and steeper upward slopes significantly reduced the step cycle delay between the hindlimb and its homolateral forelimb (Figures 5C–5E). Effect magnitude and absolute phase depended on left-right coordination, such that homolateral phase was, on average, 0.2–0.4 π rad more synchronized, and the effects of snout-hump angle or slope were 0.05–0.20 π rad smaller, when the corresponding hindlimb step was right-leading rather than left-leading or alternating (Figures S8B–S8D). Speed also showed a stronger influence on the homolateral phase, both directly and through its interaction with snout-hump angle, compared to head-fixed conditions (Figure 5F). Still, its interaction with surface slope, although statistically significant, remained below 0.1 π rad across the full 80° range of tested slopes (Figure 5G), consistent with the largely speed-independent slope effect observed on the passive treadmill. Overall, given that the absence of head fixation prevented changes in total leg load, these results support the hypothesis that anteroposterior load distribution is a key modulator of homolateral coordination. That the inclusion of trial type as a predictor eliminated the apparent differences in biomechanical effects between head height and slope trials, further strengthening this interpretation (Figure 5H).

The most striking difference between motorized and passive treadmill experiments is that the effects of snout-hump angle and slope during non-restrained locomotion were comparatively small, accounting for only 22–24% of the phase shift observed on the passive treadmill. Notably, within the range of recorded snout-hump angles, there was no evidence of a sharp, sigmoid-like transition in the homolateral phase, such as the one seen around 160° on the passive treadmill (compare Figures 3B and 5C). This apparent reduction in homolateral phase modulation by biomechanical variables could be explained by a number of factors. First, the homolateral phase was more strongly modulated by hindlimb phase, including a significant difference between left-leading and right-leading strides (level trials: $HPD_{\mathrm{SSDO}}$ = (0.89,1.01), slope trials: $HPD_{\mathrm{SSDO}}$ =(0.92–1.10)). The correlation between homolateral and hindlimb phase was also approximately twice as large on the motorized treadmill (circular-circular correlation coefficient: 0.39–0.40) as on the passive treadmill (0.16–0.17), suggesting that non-restrained, non-stimulated locomotion relied more on interlimb coupling than on biomechanical feedback. Second, without changes in the total leg load, the relative shift in load along the anteroposterior body axis was probably less pronounced. Third, mice countered increases in surface slope by both adopting more hunched postures and positioning their feet more anteriorly (Figures S8E and S8F). This combined strategy likely mitigated the posteriorward load redistribution imposed by inclined surfaces more effectively than the adaptations mice used on the passive treadmill. Finally, due to the variability in head pitch angle during non-restrained locomotion, the snout-hump angles recorded under those conditions did not directly correspond to the snout-hump angles observed during head fixation (Figure S8G). This discrepancy could have diluted the phase modulation effect attributed to snout-hump angle, making it more challenging to relate homolateral phase shifts to leg load distribution. In fact, given that mice tended to tilt their head downward on average, it is plausible that their load distribution remained sufficiently anterior-biased to confine the homolateral phase within the saturation region typically associated with smaller snout-hump angles on the passive treadmill. At the same time, it is plausible that load redistribution along the anteroposterior body axis accounts for only part of the observed effect on the homolateral phase, with the remainder driven by changes in total load that were absent in non-restrained conditions. Since total load and its distribution showed opposite relationships with snout-hump angle and slope (see panels E and G in Figure 1), influences from both of these load-related variables could also contribute to the smaller effect attributed to changes in slope on the passive treadmill. Nevertheless, our results during self-initiated, non-restrained locomotion are consistent with the anteroposterior load distribution having a role in homolateral coordination, even if the observable effect is smaller.

## Discussion

We have shown that homolateral limb coordination in mice strongly relates to the distribution of leg load along the anteroposterior body axis. Specifically, greater relative loading of the hindlimbs is associated with a largely speed-independent shift in homolateral phase preference from strict alternation toward up to a quarter-of-phase more synchronized coordination. This load-dependence was not observed for left-right (hindlimb) coordination (see “Supporting analysis of hindlimb coordination” in supplemental information), highlighting the robustness of the homolateral effect. Importantly, our conclusions are limited to changes in limb phase, rather than canonical gait identities.

The link between leg load distribution and homolateral coordination aligns with several previous findings. For example, the baseline weight distribution of mice, corresponding to a center of support near −0.1 cm,^38^ favors strict homolateral alternation, characteristic of the trotting gait. This pattern holds for most quadrupeds, except in species with markedly fore- or hind-biased weight distributions, such as giraffes or ring-tailed lemurs, that bypass trotting and transition directly from walk to canter.^5^^,^^39^^,^^40^ Moreover, the magnitude of the observed homolateral phase shift upon surface slope manipulation is similar to earlier work in opossums that reported a ∼0.16 π phase difference between 30° inclines and declines.^16^ These results are consistent with the established view that trot provides high mechanical stability through diagonal support, but maintaining this gait requires continuous limb repositioning and load redistribution along the anteroposterior body axis.^41^ Highly uneven load distributions, either due to animal morphology or environmental factors such as slope, likely constrain the range of load adjustment available and make transitions to more temporally dispersed gaits mechanically necessary. It is also worth noting that load-dependent changes in homolateral coordination occurred irrespective of hindlimb phase and during head-fixed locomotion, where the risk of falling was eliminated. This suggests that the benefits of adjusted homolateral timing may extend beyond stability, possibly reducing energetic cost via more distributed footfall impacts.^15^^,^^42^

**Figure 6:**
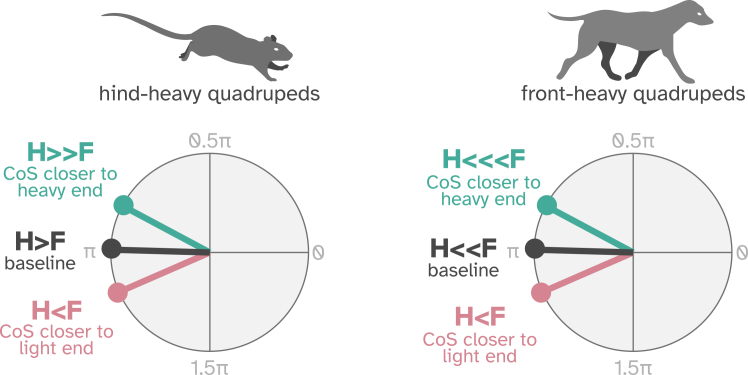
Proposed link between anteroposterior load distribution and the homolateral phase Homolateral coordination in quadrupeds may be determined not by the absolute load distribution, but by its deviation from the species’ baseline. Hindlimb phase lag decreases as the center of support (CoS) shifts toward the limb pair bearing more weight at rest (hind in mice, fore in dogs), and extends beyond π when the CoS shifts toward the pair less loaded at baseline (fore in mice, hind in dogs).

However, the direction of the observed load-related shift in homolateral coordination, namely a decrease in forelimb phase lag at greater hindlimb loading, contradicts findings from studies in opossums and dogs, where a shorter forelimb phase delay has accompanied more fore-biased load distributions, such as those associated with declines or added pectoral weight.^14^^,^^15^^,^^16^^,^^43^ This discrepancy may reflect differences in baseline load distributions. Most quadrupeds, including dogs and opossums, bear more weight on their forelimbs, while mice carry a greater proportion of weight on their hindlimbs.^38^^,^^39^ In fact, the most posterior load distribution tested in dogs resembles the most front-heavy conditions experienced by mice here (∼63% forelimb load), and these conditions have been linked to a slight increase in homolateral phase lag beyond strict alternation (1.02–1.10 π rad) in both species. Similarly, it is in more forelimb-loaded conditions of front-heavy animals and more hindlimb-loaded conditions of hind-heavy animals that the homolateral phase lag is reduced. These findings suggest that homolateral coordination may depend less on the absolute load distribution and more on the direction of its deviation from species-specific baseline values (Figure 6).

Another question to consider is how these findings relate to canonical quadrupedal gaits, such as walk, trot, gallop, and bound. It is tempting to liken the concerted, load-dependent changes in homolateral phase and limb support patterns to transitions between diagonally supported trot and three-limb-supported walk during hindlimb alternation, or between trot and distributed-support gallop during asymmetric hindlimb coordination. However, throughout the article, we have intentionally refrained from interpreting these results as transitions between canonical gaits, focusing instead on individual limb pairs and treating interlimb coordination as a continuous phenomenon. This approach was motivated by the continuous variability of interlimb coordination evident in our data and reported previously in mice, fruit flies, and horses,^44^^,^^45^ and it allowed us to identify a continuous biomechanical influence on the homolateral phase. The persistence of this influence across hindlimb phases, speeds, and axes of support variability highlights the robustness of this effect, at least under feedback-dominated locomotor conditions. In fact, during motorized treadmill locomotion, characterized by tighter coupling of homolateral and left-right phases, the effects of biomechanical variables were substantially reduced. While some of this attenuation may reflect the limited scope for weight redistribution under non-restrained conditions, it is also possible that our two experimental paradigms —one involving head fixation and optogenetic stimulation, the other imposing speed through a motorized belt— represent distinct points along a continuum from feedback-to feedforward-dominated control.^46^ In this context, it is also interesting to note the modest, but significant and highly speed-dependent, influence of total load on hindlimb coordination (see section “Supporting analysis of hindlimb coordination” in supplemental information), which suggests that, under strong descending drive from CnF stimulation, speed-dependent changes in hindlimb phase emerge primarily at higher loads. This apparent competition between descending and biomechanical control aligns with theoretical predictions that sensory feedback exerts greater influence on interlimb coordination at lower speeds, when top-down input is weaker.^17^^,^^47^^,^^48^ Further studies using non-stimulated head-fixed locomotion or varying stimulation intensities could help clarify the relative roles of descending drive, load, and feedforward control of limb coupling in interlimb coordination.

Still, the development of a locomotor paradigm that reliably exposes the role of body biomechanics in interlimb coordination, using an experimentally tractable species, is a major contribution of this study. Previous methods for modulating leg load, such as swim tests or alterations to surface texture, have been limited to binary or discrete load changes,^31^^,^^49^^,^^50^ while adding extra weight is impractical for small animals such as mice.^11^^,^^14^^,^^41^ Similarly, manipulating slope during non-restrained locomotion allows compensatory postural adjustments that counteract load redistribution, likely reducing interlimb phase shifts needed for stable movement.^13^^,^^16^^,^^32^ Consequently, such approaches have generally produced minimal or inconsistent effects on interlimb coordination and have been insufficient to separate the roles of total load and its distribution.^9^^,^^11^^,^^14^^,^^15^^,^^16^ Moreover, the lack of precise biomechanical control has made it difficult to dissociate primary effects on interlimb phase from secondary effects driven by changes in speed.^26^ By combining head fixation with the manipulation of head height or surface slope, this study overcomes the aforementioned challenges, offering continuous control over load distribution across an extended range. In addition, the use of CnF stimulation has made the paradigm more effective than traditional motorized treadmill setups at evoking diverse interlimb coordination patterns, necessary for studying changes in gait. Collectively, these innovations have been pivotal in enabling the passive treadmill experiments to reveal a stronger association between homolateral phase and load distribution than observed previously during non-restrained conditions, and to statistically dissociate the expression of speed and gait. This paradigm thus opens new, behaviorally driven avenues for investigating quadrupedal gait without relying on loss-of-function interventions to reveal gait-specific effects.

### Limitations of the study

The primary limitation of the study is that the relationships between load-related variables and interlimb coordination are merely correlational, and their causality will have to be probed by future circuit-level work. Further studies will also benefit from varying the intensity of optogenetic CnF stimulation to temper the prevalence of left-right synchronized coordination and thereby enable more uniform coverage of plausible homologous limb phases. Another notable limitation of the work is our inability to measure leg loads on the passive treadmill and during locomotion in general. Given the distinct postural adaptation strategies used by mice on the load sensors and the passive treadmill, we were unable to verify that the relationships between CoS and the snout-hump angle or slope were equivalent on the two experimental setups. Lastly, there is a significant trade-off between the experimental control provided by the passive treadmill paradigm and preservation of natural locomotor kinematics. Head fixation prevents rotational head movement, eliminates the possibility of falls, and limits the vertical body oscillation seen in normal locomotion. Although the motorized treadmill was intended as a non-restrained control, inherent kinematic differences between locomotion on stationary and mobile surfaces limited its utility. To further elucidate the mechanisms linking biomechanical changes to shifts in limb phase, future studies could track leg joints and spine curvature in addition to foot and hump positions. However, both optogenetic stimulation and head fixation were essential components of the experimental design, as they enabled a wide spectrum of interlimb coordination patterns, an expanded range of load distributions, and manipulation of total load. Furthermore, given our focus on high-speed locomotion conducive to trot-to-gallop transitions, motorized treadmill locomotion provided a more suitable and practical control than overground locomotion.

## Resource availability

### Lead contact

Further information and requests for resources should be directed to and will be fulfilled by the Lead Contact, Zane Mitrevica (zane.mitrevica.15@alumni.ucl.ac.uk).

### Materials availability

This study did not generate new materials.

### Data and code availability

• Data: Data have been deposited on Figshare and are publicly available at https://doi.org/10.6084/m9.figshare.30735518.
• Code: All original code has been shared on GitHub at https://github.com/zanemit/sensory-dependent-gait.
• Additional information: Any additional information required to reanalyse the data reported in this article is available from the lead contact upon request.

## Acknowledgments

This work was funded by a Sainsbury Wellcome Center Core grant from the 10.13039/501100000324Gatsby Charitable Foundation (219627/Z/19/Z to A.J.M.) and a Sainsbury Wellcome Centre PhD studentship (to Z.M.). We thank F.Claudi, F.Marbach, E.Chong, A.Akrami, R.Brownstone, M.R.Carey, M.W.Mathis, M.Stephenson-Jones, and members of the Murray and Branco laboratories for discussions and training, and are grateful to T.Branco for comments on the article and extensive support.

## Author contributions

Conceptualization, Z.M. and A.J.M.; methodology, Z.M.; investigation, Z.M.; formal analysis, Z.M.; writing – original draft, Z.M.; writing – review and editing, Z.M. and A.J.M.; funding acquisition, A.J.M. and Z.M.; resources, A.J.M.; supervision, A.J.M.

## Declaration of interests

A.J.M. is a co-founder and co-CEO of Antelope Health.

## STAR★Methods

### Key resources table

**Table:** 

REAGENT or RESOURCE	SOURCE	IDENTIFIER
**Antibodies**		

Goat anti-ChAT	Millipore	Cat# AB144P; RRID: AB_2079751
Alexa Fluor® 594 AffiniPure Fab Fragment Donkey Anti-Goat IgG (H + L)	Jackson Immunoresearch	Cat# 705-587-003; RRID: AB_2340435

**Bacterial and virus strains**		

AAV1-EF1a-DIO-hChR2(H134R)-EYFP	Addgene	Cat# 20298; RRID: Addgene_20298

**Deposited data**		

Data to regenerate figures	Figshare	https://doi.org/10.6084/m9.figshare.30735518

**Experimental models: Organisms/strains**		

Mouse: C57BL/6J	Charles River	Strain code: 027
Mouse: Vglut2-ires-Cre	Jackson Laboratory	JAX: 016963; RRID: IMSR_JAX:016963

**Software and algorithms**		

Custom code for data analysis and visualization (Python and R)	GitHub	https://github.com/zanemit/sensory-dependent-gait
Bonsai	Lopes et al. 2015^51^	https://bonsai-rx.org
DeepLabCut	Mathis et al. 2018,^52^ Nath et al. 2019^53^	https://github.com/DeepLabCut/DeepLabCut

**Other**		

Low-Friction Rodent-Driven Belt Treadmill	Jackson et al. 2018^54^	Janelia 2017-049

### Experimental model and study participant details

#### Animals

All animal experiments were performed under the UK Animals (Scientific Procedures) Act of 1986 Project License (PPL) PE4FA53CB, following approval by the Animal Welfare and Ethical Review Body of Sainsbury Wellcome Center, UCL. Male and female mice (at least 40% of each sex) were housed on a reversed 12 h light-dark cycle with *ad-libitum* access to food pellets and water. The passive treadmill and load sensor experiments were done with 10–20 weeks old Vglut2-ires-Cre mice (Jackson Laboratory, stock 016963). The motorised treadmill experiments used 6–9 weeks old Vglut2-ires-Cre and C57BL/6J wild type (Charles River 027) mice. Animals were weighed at least once a week during all behavioral procedures. Animal sex had no significant influence on the key homolateral phase effect reported in this manuscript ($HPD_{\mathrm{SSDO}}$=(-0.02,0.48)).

### Method details

#### Viruses

For optogenetic triggering of locomotion, 23 nL of AAV1-EF1a-DIO-hChR2(H134R)-EYFP (titer 1.0×10^13^, Sainsbury Wellcome Center Viral Vector Core, henceforth SWC VVC, from Addgene plasmid 20289) was injected unilaterally into the cuneiform nucleus (CnF) of vGlut2-Cre mice at AP -5.0, ML 1.2, DV -3.0 mm from bregma. Control mice received the same injection but were of the C57BL/6J phenotype. These mice had no perceptible reaction to the optical stimulus and were not used for data acquisition.

A subset of vGlut2-Cre and wild type mice used in the motorised treadmill experiments had received a bilateral CnF injection of 23 nL AAV2-EF1a-DIO-iChloc-2A-dsRed (titer 1.1×10^14^, SWC VVC from Addgene plasmid 70762) for a separate experiment, but showed no discernible reaction to the optical stimulus, did not differ in their locomotor performance (one-way ANOVA, *p* = 0.29), and were therefore pooled for analysis.

#### Surgical procedures

Mice were anesthetized with 4% isoflurane and maintained under anesthesia for the duration of the surgery with 1.5–2.5% isoflurane (both in oxygen 1 L/min). Analgesia was provided through subcutaneous administration of meloxicam (5 mg/kg).

Viral vectors were delivered via pulled 3.5″ glass pipettes (Drummond scientific) at a speed of 23 nL/s with a Nanoject II injector (Drummond scientific) coupled to a stereotaxic frame (Kopf model 902). Optic fiber cannulae (New Doon, FOC-C-1.25-200-7.0-0.37) and stainless steel headplates were affixed to the skull using a combination of light-cured (RelyX Unicem 2, 3M) and self-curing (Superbond, C&B) dental cement.

#### Histological processing

At the end of all behavioral experiments, mice were anesthetized with intraperitoneally injected pentobarbital (Euthatal or Dolethal 30 μg/kg) and transcardially perfused with 10–15 mL of 0.01 M phosphate buffered saline (PBS) followed by 4% paraformaldehyde solution in PBS. Brains were post-fixed at room temperature overnight and cut into 50 μm coronal sections on a vibratome (Leica VT1000 S).

To locate the boundary between the target region (CnF) and its neighboring pedunculopontine nucleus (PPN), brain slices were immunohistochemically stained for choline acetyltransferase (ChAT). Sections were first permeabilised and blocked in a solution containing 1% bovine serum albumin (BSA, Cambridge Bioscience) and 0.3% Triton X-(VWR International Ltd) in 0.01 M PBS for 1.5 h at room temperature, on a shaker. In parallel, goat anti-ChAT (Millipore AB144P, 0.1 mg/mL, 1:100 dilution) was pre-incubated with an anti-goat Fab fragment (Jackson Immunoresearch AF594, 1.6 mg/mL) in a solution containing 1% BSA and 0.1% Triton in 0.01 M PBS for 1.5 h at 37°C The sections were subsequently incubated in this primary antibody solution for 3 days at 37°C on a shaker. This was followed by three 30-min washes with the 1% BSA, 0.1% Triton PBS solution and a final 30-min wash with PBS. Finally, the sections were counterstained and mounted with DAPI Fluoromount-G (Southern Biotech), and imaged at 1.3 μm/px resolution on an epifluorescent microscope (Zeiss Axio Imager 2).

#### Passive treadmill locomotion

After recovery from surgery, mice were habituated over 10 days to the passive treadmill setup, adapting to head fixation, blue light exposure, and changes in head elevation and surface slope. The duration of daily habituation sessions increased progressively from 15 to 50 min. During each session, head-fixed mice remained stationary or voluntarily locomoting on a low-friction, non-motorised treadmill constructed according to the documentation and source code released by the Janelia Research Campus.^54^ Depending on the planned experimental conditions, head height, surface slope, or both were adjusted multiple times per session. To reduce stress, small drops of condensed milk were delivered at random intervals. In the final three sessions, animals were gradually exposed to low-intensity blue light flashes from above to adjust to the visual component of the upcoming optogenetic stimulation.

Following the habituation period, 5 s optical stimuli (10–50 Hz, 10 mW at fiber tip) were delivered with a 473 nm laser (Shanghai Laser & Optics Century) for 20–25 trials daily to induce locomotion. Only the mice that showed reliable locomotor responses to the stimulation were used in the experiments. Videos of the left and right side views were recorded at 400 frames per second using a pair of cameras (Basler acA1920-150um). Data acquisition, camera trigger, and optogenetic stimulation were synchronised through a PCIe-6351 board (National Instruments) and controlled using Bonsai.^51^

Biomechanical feedback to the animal was manipulated with two types of trials. In head height trials, the head-fixation apparatus was adjusted between 32 and 57 mm above ground with ±0.1 mm precision while keeping the surface slope fixed at 0°. The setup did not allow head rotation along any axis. In surface slope trials, the treadmill, head-fixation apparatus, and cameras were jointly tilted in 5±1 degree increments over a [-40, 40] degree range, while keeping the head fixed at a 44 mm height. These same ranges of head heights and surface slopes were used during both habituation and data collection.

Locomotor analysis was performed only on the mice that performed at least 1000 strides across experimental days and conditions.

#### Leg load measurements

Headplated vGlut2-Cre mice were head-fixed on a custom setup of four single-point aluminum load cells (Tedea Huntleigh, dimensions: 11 × 3.3 × 1 cm), arranged so that each cell supported exactly one foot, as in.^38^ Like the passive treadmill, the force sensor setup enabled independent manipulation of head height and surface slope. In head height trials, head-fixation height was varied between 32 and 57 mm above ground with ±0.1 mm precision. In slope trials, the whole apparatus was tilted between −40° and 40° while keeping the head fixed at 44 mm.

Load cells were calibrated ahead of each recording session. Load measurements were amplified 250000x and high-pass filtered at 100 Hz (pre-amplifier MA103S and amplifier MA102S, both custom-built in the Neuroscience Electronics Lab, University of Cologne), digitised at 1428 Hz (Cambridge Electronic Design Power1401), and recorded in Spike2 software (Cambridge Electronic Design). In parallel, videos with the right side view of the mouse were acquired at 100 Hz using Bonsai.^51^ Only trials with mice standing still for at least 5 s were included in analysis. Horizontal and fore-aft force components could not be measured with this setup.

#### Motorised treadmill locomotion

Experiments began with a 15-min habituation session. During this session, mice were allowed to freely explore a custom-built motorised treadmill (model 009, Neuroscience Electronics Lab, University of Cologne) and were exposed to three 30-s bouts of treadmill movement at 5 cm/s. This was followed by up to 10 days of treadmill training. Each day, unrestrained mice performed 8–12 locomotor trials lasting 15 s each. Treadmill speed was progressively increased from 15 cm/s up to 150 cm/s based on individual performance, using Spike 2 software (Cambridge Electronics Design). The treadmill’s speed output was used to trigger closed-loop video acquisition via a PCIe-6351 board (National Instruments) and Bonsai.^51^ High-speed videos (400 fps) were captured from both left and right sides using two Basler acA1920-150um cameras.

#### Sound-triggered escape locomotion

To observe natural high-speed locomotion, we designed an elevated open field arena (20 × 60 cm) from a transparent acrylic platform with 10 cm tall walls. Animal behavior was recorded from below at 60 frames per second using an industrial camera (Basler acA1920-150um). A shelter was placed along the short wall, and mice were exposed to threatening auditory stimuli to evoke escape responses. In particular, prior to experiments, wild-type mice were single-housed to increase their baseline stress level. During each experimental session, the mice were first allowed to explore the arena unperturbed for 7 min. Subsequently, when mice entered a threat area located ∼10 cm from the wall opposite the shelter, they were pseudorandomly exposed to an aversive auditory stimulus composed of three consecutive upsweeps from 17 to 20 kHz over 3 s.^55^ Each session lasted up to an hour but was terminated earlier if the animals did not leave the shelter for 20 min.

### Quantification and statistical analysis

#### Tracking of body parts

Tracking of the four feet, snout, tail base, and either the hump (side view) or body center (bottom view) was performed using DeepLabCut (version 2.1.10.4^52^^,^^53^). We labeled 20 frames from 72 videos (passive treadmill, each side-view camera separately), 48 videos (load sensor head height trials), 27 videos (load sensor slope trials), or 50 videos (motorised treadmill, each side-view camera separately), and used 95% of those for training. Neural networks were trained for 650000 iterations using the default parameters and ResNet-101 as a starting point, and validated with 10 shuffles. The train and test errors were 0.34±0.01 mm and 1.97±0.20 mm respectively (passive treadmill, right side view), 0.26±0.02 mm and 1.73±0.22 mm (passive treadmill, left side view), 0.55±0.05 mm and 0.80±0.16 mm (load sensors, head height trials), 0.28±0.02 mm and 0.65±0.07 mm (load sensors, slope trials), 0.56±0.02 mm and 1.55±0.13 mm (motorised treadmill, right side view), 0.48±0.02 mm and 1.49±0.16 mm (motorised treadmill, left side view), 0.84±0.02 and 2.18±0.61 (open field, bottom view). For reference, the body of an average mouse took up approximately 65 × 20 mm.

Included in further analyses were only those treadmill trials where at least 90% of the predicted ‘snout’ and ‘hump’ coordinates and at least 80% of the predicted ‘left hindlimb’ (primary reference limb) coordinates had a tracking likelihood equal to, or greater than, 0.95. The predicted body part coordinates were processed with custom Python scripts to extract postural and kinematic variables, including snout-hump angle, locomotor speed, and interlimb phase.

#### Quantifying body weight distribution

Body weight distribution across head heights and surface slopes was estimated by combining load cell outputs with body weight measurements taken within three days of each recording session and interpolated accordingly. In the absence of head fixation, the total load recorded by all four load cells would be expected to equal the animal’s body weight. However, head fixation introduced discrepancies between these two measures due to the tensile or reactive force arising from interaction with the head fixation apparatus. If the total recorded load was lower than the animal’s body weight, it suggested partial offloading onto the head fixation apparatus. In contrast, readings exceeding body weight likely reflected not only gravitational load but also upward reactive forces generated by the animal pushing against the headbars.

In slope trials, because only the vertical component of ground reaction force was recorded, the detectable body weight was reduced to cos(θ)⋅weight, where θ is the incline or decline angle. This scaling was taken into account when computing the fraction of body weight transferred onto the head fixation apparatus. However, since the same scaling applied to all four load cells, slopes did not affect our computation of weight distribution across the legs.

To reduce the dimensionality of body weight distribution for analytical convenience, we used the fractions of total load $w_{i}$ recorded on each foot i to compute center of support (CoS) along the anteroposterior and mediolateral body axes:

$$CoS_{AP}≔w_{RF}+w_{LF}-\left(w_{RH}+w_{LH}\right)$$

$$CoS_{ML}=w_{RF}+w_{RH}-\left(w_{LF}+w_{LH}\right)$$

where RF, RH, LF, and LH represent load cell measurements corresponding to the right forelimb, right hindlimb, left forelimb, and left hindlimb respectively. Slopes and interactions with the head fixation apparatus were irrelevant for this metric.

#### Computing weight-adjusted head height

To account for body size-related variation in the relationship between head height and load distribution, we introduced a dimensionless metric called weight-adjusted head height. It was assumed that maximum comfortable head height, namely the highest level at which both forefeet remain in ground contact, scaled linearly with body weight. This height was estimated empirically (*N* = 8 mice) by manipulating head height in 1 mm increments. The following relationship was derived:

Maximumcomfortableheadheight=24.5+1.25×Weight

For instance, 22 g and 27 g mice were estimated to have maximum comfortable head heights of 52 mm and 57 mm respectively.

Weight-adjusted head height was then calculated as:

$$\text{Weight adjusted}\;\text{head}\;\text{height}=\frac{\text{Physical}\;\text{head}\;\text{height}-24.5}{1.25\times \text{Weight}}$$

In other words, a weight-adjusted head height value of 1 represents the maximum head height at which a mouse of a given weight had its forefeet in full contact with the ground.

In surface slope trials, all mice were head-fixed at 44 mm above the ground, which was near the middle of the comfortable head height range. For animals of different sizes, this absolute head height corresponded to weight-adjusted head heights between 0.62 and 0.68. Because weight-adjusted head height was significantly associated with load-related variables, using a fixed head height across animals contributed to inter-individual variability in leg load recordings. Tailoring head height to each animal’s weight would have likely reduced this variability. Still, this limitation is unlikely to affect the main conclusions, as the variation in weight-adjusted head height across mice was relatively low (coefficient of variation: 3% and 11% on force sensors and treadmill respectively) and the leg load variability was similar in head height and slope trials (*p* = 0.68, *t test*). Moreover, instead of head height, the majority of analyses relied on the snout-hump angle, which served as a weight-independent proxy for posture.

#### Regression analysis of non-circular data

Linear relationships between non-circular variables were analyzed using simple linear mixed-effects regression models that included either a random intercept (y∼x+(1|mouse)) or both a random intercept and a random slope (y∼x+(x|mouse)). Models were fitted using the lmer function from the lme4 package (v1.1.28,^56^) in R. Inclusion of random effects allowed us to capture inter-animal variability in model parameters. To verify that linear model assumptions were satisfied, model residuals were examined for normality using QQ plots and for homoscedasticity by plotting residuals against fitted values. While minor deviations from normality occurred at the extremes, they affected only a small number of observations and their temporary exclusion did not substantially affect the results. The significance of fixed-effect predictors was tested using Student’s t-tests with Satterthwaite’s approximation of degrees of freedom as implemented in the lmerTest package (v3.1.3). Thresholds of p < 0.05, p < 0.01, p < 0.001 were applied and indicated by asterisks in figures. When models including a random slope did not significantly improve fit compared to random intercept-only models, we nevertheless retained the random slope structure to obtain more conservative estimates of fixed-effect significance. For analyses involving multiple linear predictors, this approach was extended to multiple mixed-effects regression.

In cases where the data exhibited saturating trends, an exponential decay function was fit:

$$y=A-Be^{-kx}$$

Here, y is the outcome variable, x is the predictor, A is the asymptote, B is the scale factor, and k is the rate constant. Initial parameters were estimated using empirical heuristics: A from the observed maximum, B from the difference between initial y and A, and k as the inverse of the mean of predictor values. Curve fitting was performed separately for each mouse using the curve_fit function from the scipy.optimize library in Python. Because of the relatively small number of animals and the non-normality of parameter distributions, statistical significance of group-level parameter effects was assessed using Wilcoxon signed-rank tests.

#### Interlimb phase computation

Interlimb coordination was quantified through the phases of limb movement relative to a reference limb: the right or left hindlimb for right or left homolateral phase respectively, the left hindlimb for hindlimb phase, and the left forelimb for forelimb phase. Where data was pooled across right and left homolateral phases, the term ‘homolateral phase’ is used without reference to body side. When phase could not be computed in absolute terms because data was pooled across distinct trial types (Figure 3H), we instead calculated the ’homolateral phase shift’, namely the change in homolateral phase relative to its value at one extreme of the independent variable.

All phase calculations were based on the time series of tracked foot positions, focusing on the time periods where the speed of the self-paced or motorised treadmill belt exceeded 1 cm/s indicative of locomotion. In particular, the horizontal trajectory of the reference foot was segmented into strides by applying a peak detection algorithm. Peaks and troughs marked swing and stance onsets respectively, with strides defined as the intervals between consecutive swing onsets. The last stride in each locomotor bout was truncated at the final swing onset. To compute the relative phase of each non-reference limb, its horizontal trajectory was cross-correlated with that of the reference limb for every reference limb stride of duration d over delays in range [$\frac{-d}{2}$, $\frac{d}{2}$]. The temporal lag corresponding to the peak of this cross-correlation reflected the interlimb phase difference. It was normalised by stride duration to yield a value in interval [−0.5,0.5] (or [−π,π] in polar coordinates). This procedure was applied to two independently tracked points on each foot. If their phase values differed by more than 0.1 π, the particular stride was excluded from analysis.

Given the frequent absence of clear boundaries between the observed combinations of limb phases,^44^ the analysis refrained from assigning discrete gait labels to individual strides and instead considered limb phase distributions under various conditions. Average phase distributions were quantified using von Mises kernel density estimation (κ = 10, 200 bins), with hyperparameters chosen from a tested range (κ = [5,15], 60–300 bins) to balance resolution and smooth appearance. Data from mice that performed fewer than 20 strides in a given category were excluded. Other analyses did not exclude data on the basis of sparseness.

#### Circular mixture model analysis of phase data

The distribution of relative limb phases, including its modality, was analyzed using univariate Von Mises mixture models (1–4 components) implemented in the BAMBI R package.^57^ For each subset of data, such as specific snout-hump angle ranges, model selection was guided by Watanabe-Akaike information criterion (WAIC) and Z-tests applied to the differences in expected log predictive density. All models were fit with three Markov chains, 5000 burn-in iterations, and 5000 sampling iterations.

#### Circular-linear regression of phase data

To quantify how limb phase relates to kinematic, postural, and environmental factors, while accounting for inter-animal variability, we applied a Bayesian mixed-effects regression framework for circular data using the bpnreg package in R (version 2.0.2,^58^).

This analysis assumes unimodal, normally distributed circular data, and independence of observations. To satisfy the unimodality assumption, data from a given mouse was included in the analysis only if the corresponding best-fit Von Mises mixture model met one of the following criteria: (1) it had a single component, (2) the dominant component accounted for at least 80% of the model, or (3) two components jointly explained at least 80% of the data and differed in mean direction by less than 0.2π. Since each data point represented a stride defined by consecutive swing onsets of the reference limb, the independence assumption was not strictly met. We assessed potential autocorrelations by including stride number within a locomotor bout as a predictor, accepting the model if its effect was statistically insignificant or negligible (≤0.01π per stride). When datasets remained large (>10,000 observations) after balancing, we further mitigated autocorrelations by fitting models to random subsamples of 30–70% of the data. However, a small degree of autocorrelation could still contribute to the narrow HPD intervals we observed.

All models involved limb phase as the circular response variable, and random intercept for mouse identity accounted for repeated observations per subject. Linear predictors, their interactions, and random slopes varied by model as described below using R-style formula notation, with angle referring to snout-hump angle and reference limb ID referring to whether the homolateral phase was computed on the left or right side of the body. Note that effects attributed to reference limb identity describe the average differences in left and right homolateral phase across strides, not instantaneous differences within a stride. Models that include slope as a predictor were fit to data from surface slope trials. The only exception is models that additionally include trial type as a predictor; those were fit to combined data from head height (or level) and slope trials.

• The following regression models were fit to passive treadmill strides with hindlimb phase treated as a categorical predictor: left-leading or right-leading.
  ○ Figure S4B: Left homolateral phase ∼ speed ∗ angle ∗ right hindlimb phase category + (angle | mouse ID).
• The following regression models were fit to passive treadmill strides with hindlimb phase treated as a categorical predictor: alternating, synchronised, or asymmetric (pooled left-leading and right-leading).
  ○ Figure S4C: Left homolateral phase ∼ speed ∗ angle ∗ right hindlimb phase category + (angle | mouse ID).
  ○ Figures S4G and S4H: Left homolateral phase ∼ speed ∗ angle ∗ slope ∗ right hindlimb phase category + (angle + slope | mouse ID).
• The following regression models were fit to motorised treadmill strides with hindlimb coordination treated as a categorical predictor: alternating, left-leading, or right-leading.
  ○ Figure S8B: Left homolateral phase ∼ speed ∗ angle ∗ right hindlimb phase category + (angle | mouse ID).
  ○ Figures S8C and S8D: Left homolateral phase ∼ speed ∗ angle ∗ slope ∗ right hindlimb phase category + (angle + slope | mouse ID).
• The following regression models were fit to passive treadmill data balanced for right hindlimb phase category: alternating, synchronised, or asymmetric (pooled left-leading and right-leading).
  ○ Figure 3B: Left homolateral phase ∼ speed ∗ angle + (angle | mouse ID).
  ○ Figures 3D and 3E: Left homolateral phase ∼ speed ∗ angle ∗ slope + (angle + slope | mouse ID).
  ○ Figure S4K: Left homolateral phase ∼ speed ∗ angle ∗ slope + trial type + (angle + slope | mouse ID).
  ○ Figure S4D and S4E: Homolateral phase ∼ speed ∗ angle + reference limb ID + (angle | mouse ID).
  ○ Figures 3E, S4I and S4J: Homolateral phase ∼ speed ∗ angle ∗ slope + reference limb ID + (angle + slope | mouse ID).
  ○ Figure S4L: Homolateral phase ∼ speed ∗ angle ∗ slope + weight + (angle + slope | mouse ID).
  ○ Figure S7A: Left homolateral phase ∼ stride length ∗ angle ∗ slope + trial type + (angle + slope | mouse ID).
  ○ Figure S7B and S7E: Homolateral phase ∼ duty factor ratio ∗ angle ∗ stride length + reference limb ID + (angle + slope | mouse ID).
  ○ Figure S7D: Left homolateral phase ∼ duty factor ratio ∗ angle ∗ slope + trial type + (angle + slope | mouse ID).
  ○ Figure S7C and S7F: Homolateral phase ∼ duty factor ratio ∗ angle ∗ slope ∗ stride length + reference limb ID + (angle + slope | mouse ID).
• The following regression models were fit to motorised treadmill data balanced for right hindlimb phase category: alternating, left-leading, or right-leading.
  Figure 5C: Left homolateral phase ∼ speed ∗ angle + (angle | mouse ID).
  ○ Figure 5D and 5E: Left homolateral phase ∼ speed ∗ angle ∗ slope + (angle | mouse ID).
• The following regression models were fit to passive treadmill data balanced for left forelimb phase category: alternating (0.8–1.2 π rad) or advanced (0.2–0.8 π rad).
  ○ Figure S5B and S5C: Right hindlimb phase ∼ speed ∗ angle + (angle | mouse ID).
  ○ Figures S5E–S5H: Right hindlimb phase ∼ speed ∗ angle ∗ slope + (angle + slope | mouse ID).
  ○ Figure S5I and S5J: Right hindlimb phase ∼ speed ∗ weight-adjusted head height ∗ angle residuals + (weight-adjusted head height + angle residuals | mouse ID).
• The following regression models were fit to motorised treadmill data balanced for left forelimb phase category: alternating or advanced.
  ○ Figure S5K: Right hindlimb phase ∼ speed ∗ angle + (angle | mouse ID).
  ○ Figure S5 L: Right hindlimb phase ∼ speed ∗ angle ∗ slope + (angle + slope | mouse ID).

Models were fitted with 100 burn-in iterations, 1000 output iterations, and a lag of 3. To reduce multicollinearity, predictors with a variance inflation factor exceeding 5 were excluded. In some cases, random subsamples of the data were used due to computational limitations. Model convergence was verified with traceplots for all fixed-effect predictors. Predictor significance was assessed using the highest posterior density (HPD) interval of the signed shortest distance to origin (SSDO) such that predictor was considered significant if the $HPD_{\mathrm{SSDO}}$ interval did not include zero.^58^ Model comparison was based on the Watanabe-Akaike information criterion (WAIC).

#### Quantification of left-right phase bias

To determine whether interlimb coordination was affected by the side of unilateral optogenetic stimulation, the side of optogenetic stimulation was treated as a binary outcome variable, while the sine and cosine components of the relative phase from one or multiple limb pairs were used as predictors. Data was split into train (75%) and test (25%) sets, balancing training data with respect to both the outcome variable and mouse identity. A support vector classifier was trained to classify phase data according to side of optogenetic stimulation. Statistical significance of classifier accuracy was evaluated using a permutation test with 1000 label shuffles and thresholds of p < 0.05, p < 0.01, p < 0.001 indicated by asterisks in figures. Discriminative ability was assessed using the receiver operating characteristic (ROC) curve.

#### Computation of CoS-equivalent shifts in phase

To compare limb phase shifts across surface slope and head height trials, changes in snout-hump angle or slope on the passive treadmill were assumed to produce equivalent average weight redistributions as those directly recorded on load sensors. By aligning snout-hump angles and slopes between the treadmill and force sensor setups, changes in limb phase could be mapped onto estimated shifts in anteroposterior CoS position. These CoS-equivalent shifts were calculated individually for each mouse using two data sources: (1) treadmill data from the same animals that performed both head height and slope trials, and (2) population-averaged load sensor data, derived from a separate cohort. This approach relied on the additional assumption that group-level CoS-angle relationship were typical across animals.

#### Histological quantification

Brain sections were registered to the Allen Mouse Brain Common Coordinate Framework (CCFv3,^59^) using the ABBA plugin in ImageJ (BioImaging And Optics Platform, Ecole Polytechnique Federale de Lausanne). The positions of virally labeled cells and optic fiber implants were subsequently determined in Qupath^60^ using its cell detection and manual annotation tools respectively. Finally, the data were visualised in 3D with Brainrender.^61^

## Supplemental information

Document S1. Figures S1–S8

Video S1. An example head height trial on the passive treadmill with the head fixed 52 mm above ground, related to Figure 2

Video S2. An example head height trial on the passive treadmill with the head fixed 32 mm above ground, related to Figure 2

Video S3. An example surface slope trial on the passive treadmill, inclined at 40°, related to Figure 2

Video S4. An example surface slope trial on the passive treadmill declined at 40°, related to Figure 2
