# Supplementary material for: Distinct descending and biomechanical influences on interlimb coordination in mice
Source: iScience. 2026 Feb 16;29(3):115008. doi: 10.1016/j.isci.2026.115008 (PMC12962166; doi:10.1016/j.isci.2026.115008)
Supplement: Document S1. Figures S1–S8 [file mmc1.pdf]

iScience, Volume *29*

Supplemental information

Distinct descending and biomechanical influences on interlimb coordination in mice

Zane Mitrevica and Andrew J. Murray

1 Supplemental Information

Supporting analysis of hindlimb coordination

Studying the highly interconnected locomotor control system through regression analyses is an inherently limited approach, as many variables will share variance without reflecting direct causal relationships. Still, it is worth examining biomechanical contributions to homolateral and left-right coordination separately, as differences in shared variance can help identify components that are more tightly coupled. By highlighting three key differences in load-related effects on hindlimb and homolateral coordination, these analyses support the main-text conclusion that a posteriorward load redistribution is associated with a largely speed-independent shift specifically in homolateral phase.

First, we found no systematic relationship between hindlimb phase and either snout-hump angle or surface slope. Although hindlimb coordination was, on average, near-synchronous across postural conditions and slopes (**Figure S5 A, D**), there was enough variability in hindlimb phase data (see Figure S2 G) for circular-linear regression analysis to potentially identify systematic associations between this variability and specific predictors. Still, there was no significant main effect of snout-hump angle (**Figure S5 B**) or slope (**Figure S5 E**), and phase variability was greater across animals (mean resultant length: 0.92) than observed for homolateral phase (0.99).

Second, snout-hump angle and slope showed significant interaction effects with speed. In head height trials, a significant interaction emerged between snout-hump angle and speed, such that lower speeds were associated with hindlimb desynchronisation of up to $\sim$0.9 π rad specifically when the hump-snout vector pointed steeply downward (**Figure S5 C**). In slope trials, steep inclines boosted the effect of speed in a statistically significant, albeit small ($\sim$0.06 π rad), way (**Figure S5 F**), and significant interaction effects were also observed between snout–hump angle and slope (**Figure S5 H**). Considering the relationships between biomechanical variables, load distribution, and total load (see Figure 1), these results are compatible with hindlimb phase sensitivity to speed being influenced by the total leg load, rather than its anteroposterior distribution, contrasting with the homolateral phase effect reported in the main text.

Finally, the speed-dependent effect of snout-hump angle on hindlimb phase could not be fully explained by load-related factors. Notably, the interaction between speed and snout-hump angle differed qualitatively in the slope and head height trials (**Figure S5 G**). Unlike for homolateral phase (see Figure S2 K), a circular-linear regression analysis of hindlimb phase revealed a significant effect of trial type (HPD_{SSDO} = (-0.28, -0.12)) that could not be explained by the combination of snout-hump angle and slope. To isolate this additional effect, we conducted a further circular-linear regression analysis with predictors that included speed, weight-adjusted head height, and residuals from regressing snout-hump angle against weight-adjusted head height. The latter was expected to capture variation in snout-hump angle not attributable to load. This analysis attributed significant speed-dependent effects on hindlimb phase to both snout-hump angle residuals (**Figure S5 I**) and head height (**Figure S5 J**). Moreover, the residuals produced a phase pattern similar to that of the speed-angle interaction in the slope trials, such that hindlimb synchronisation increased with speed specifically at smaller-than-predicted snout-hump angles, represented by negative residuals, while positive residuals tended to have the opposite effect. Meanwhile, weight-adjusted head height modulated the strength, but not direction, of the speed-phase relationship, aligning with slope-related effects.

Non-restrained locomotion on the motorised treadmill supported the different biomechanical effects on homolateral and hindlimb coordination. Since total load remained constant in this setting, hindlimb phase was significantly influenced by speed-angle interaction in a manner that was qualitatively consistent with the effect of snout-hump angle residuals in head-fixed trials (**Figure S5 K**), but there was no interaction between speed and surface slope (**Figure S5 L**). Together, these analyses provide supporting context for the primary findings on homolateral phase.

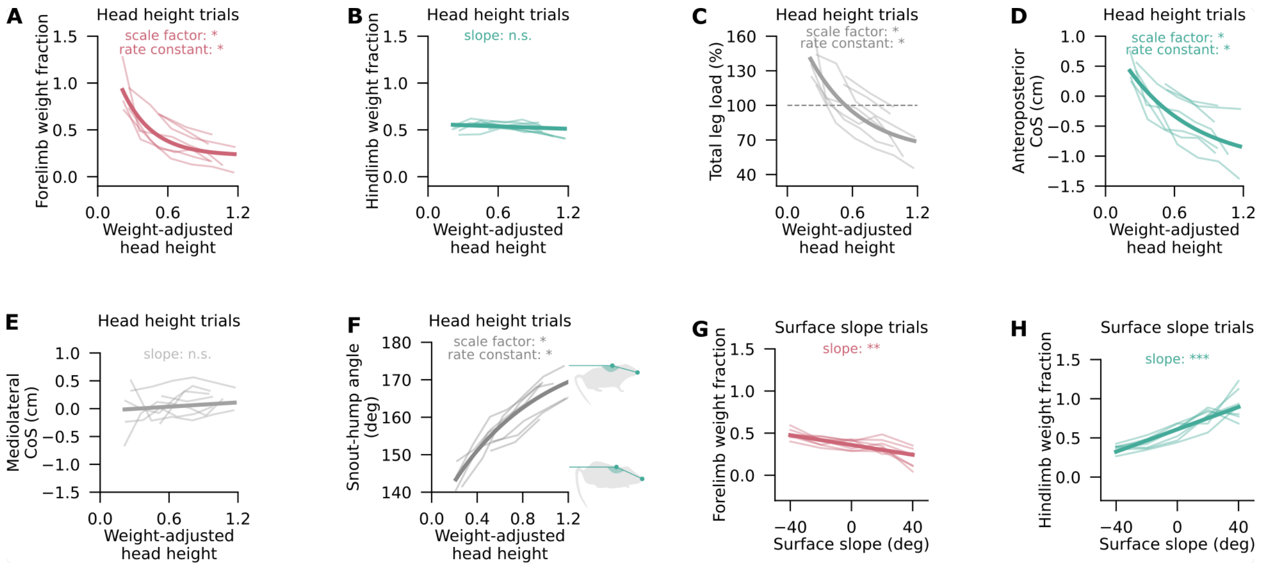

Figure S1: **Head height and surface slope modulation have distinct effects on load-related variables.** Related to Results section "A combination of biomechanical and optogenetic modulation enables systematic study of interlimb coordination".

(A-B) The fraction of body weight placed on forelimbs (A; *median scale factor: -2.0, Wilcoxon signed-rank test statistic W=0, p=0.02; median rate constant: 3.5, W=0, p=0.02*) and hindlimbs (B; *mean slope±SEM: -0.04±0.02, t-test with Satterthwaite correction t(3)=-1.9, p=0.2*) as a function of weight-adjusted head height. Shown are trial averages of individual mice (n=7; thin lines), and a function to characterise the relationship between the variables across animals (thick lines): exponential decay for forelimb weight fractions and linear fit for hindlimb weight fraction.

(C) Same as (A), but showing the total detected load (*median scale factor: -181 %, W=0, p=0.02; median rate constant: 2.1, W=0, p=0.02*) as a percentage of body weight.

(D-E) Same as (A), but showing centre of support along the anteroposterior (D; *median scale factor: -2.1 cm, W=0, p=0.02; median rate constant: 3.0, W=0, p=0.02*) and mediolateral (E; *slope: 0.2±0.1, t(5)=1.6, p=0.2*) body axes.

(F) Snout-hump angle plotted as a function of weight-adjusted head height of individual mice across trials (thin lines) and approximated by an exponential decay model (thick line; *median rate constant: 0.77, W=0, p=0.02*).

(G-H) The fraction of body weight placed on forelimbs (G; *slope: 0.0029±0.0005 per degree, t(6)=-5.9, p=0.001*) and hindlimbs (H; *slope: 0.0072±0.0008 per degree, t(6)=9.5,* $p=9\times10^{-5}$) as a function of surface slope. Shown are trial averages of individual mice (n=7; thin lines) along with the line of best fit across mice (thick).

Statistical significance refers to t-tests with thresholds: * $p < 0.05$, ** $p < 0.01$, *** $p < 0.001$.

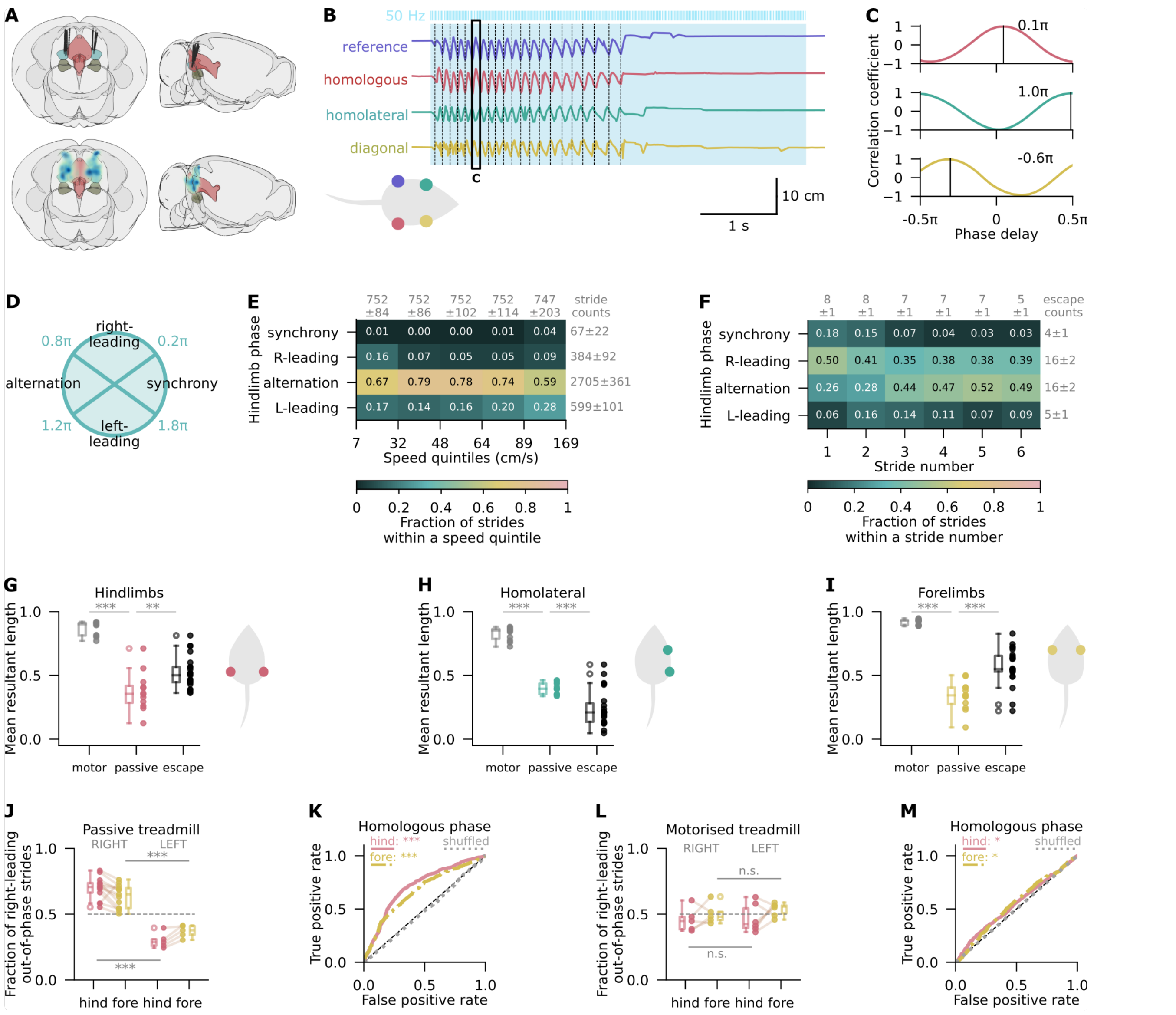

Figure S2: **The passive treadmill paradigm elicits more diverse interlimb coordination patterns than traditional locomotor approaches, but also introduces a lateral bias.** Related to Results section "A combination of biomechanical and optogenetic modulation enables systematic study of interlimb coordination".

(A) Traced positions of optic fibre implants (*top*) and density distributions of virally labelled cells (*bottom*) viewed in coronal (*left*) and sagittal (*right*) planes. CnF, PPN, and PAG are shown in blue, dark grey, and red respectively.

(B) Example x-coordinate traces of the four limbs tracked by DeepLabCut during a 50 Hz optogenetic stimulus (blue shaded region; light pulses shown by the blue bars above) and used for interlimb phase estimation. Vertical dashed lines mark swing onsets for the reference limb, defining step boundaries.

(C) Cross-correlation of the designated reference limb step from (B) and the corresponding x-coordinate traces of the three other limbs at different phase delays. Vertical lines mark the peak of the cross-correlation, used to determine the relative phase of the respective limb pair.

(D) Interlimb phase definitions used in panels E-F, Figure 2E, and elsewhere in the paper.

(E) Fraction of strides with synchronous, alternating (anti-phase), and left- or right-leading out of phase hindlimb coordination patterns across speed quintiles on the motorised treadmill. Fractions are averaged across mice and normalised within each speed quintile. Marginal stride counts, averaged across mice, are shown along rows and columns.

(F) Same as (E) but for consecutive strides in an escape bout.

(G-I) Mean resultant length of hindlimb (G), homolateral limb (H), and forelimb (I) phase distributions in motorised treadmill, passive treadmill, and escape paradigms. For reference, mean resultant length is an inverse measure of variability that varies between 0 and 1, with these extremes representing a uniform and a degenerate distribution respectively.

(J, L) The fraction of right-leading steps between hindlimbs (*pink*; p=9×10^{-8}, *t-test*) and forelimbs (*yellow*; p=2×10^{-5}) on the passive treadmill during locomotion induced by unilateral stimulation of the right or left CnF (J) or on the motorised treadmill during non-stimulated locomotion (L). Points connected by lines represent individual mice. Alternating and synchronised steps, as defined in (D), are excluded.

(K, M) Receiver operating characteristic curve of a binary classifier trained to predict the side of virus injection and optic fibre implantation based on limb phase data acquired during optogenetically stimulated (K) and non-stimulated (M) locomotion. Separate classifiers were trained on hindlimb (*pink, solid*; permutation tests with 1000 shuffles: passive p<0.001, motorised: p=0.01) and forelimb (*yellow, dash-dot*; permutation tests with 1000 shuffles: passive p<0.001, motorised p=0.004) data, and data with shuffled outcome labels (*grey, dotted*).

Statistical significance refers to t-tests with thresholds: * $p < 0.05$, ** $p < 0.01$, *** $p < 0.001$.

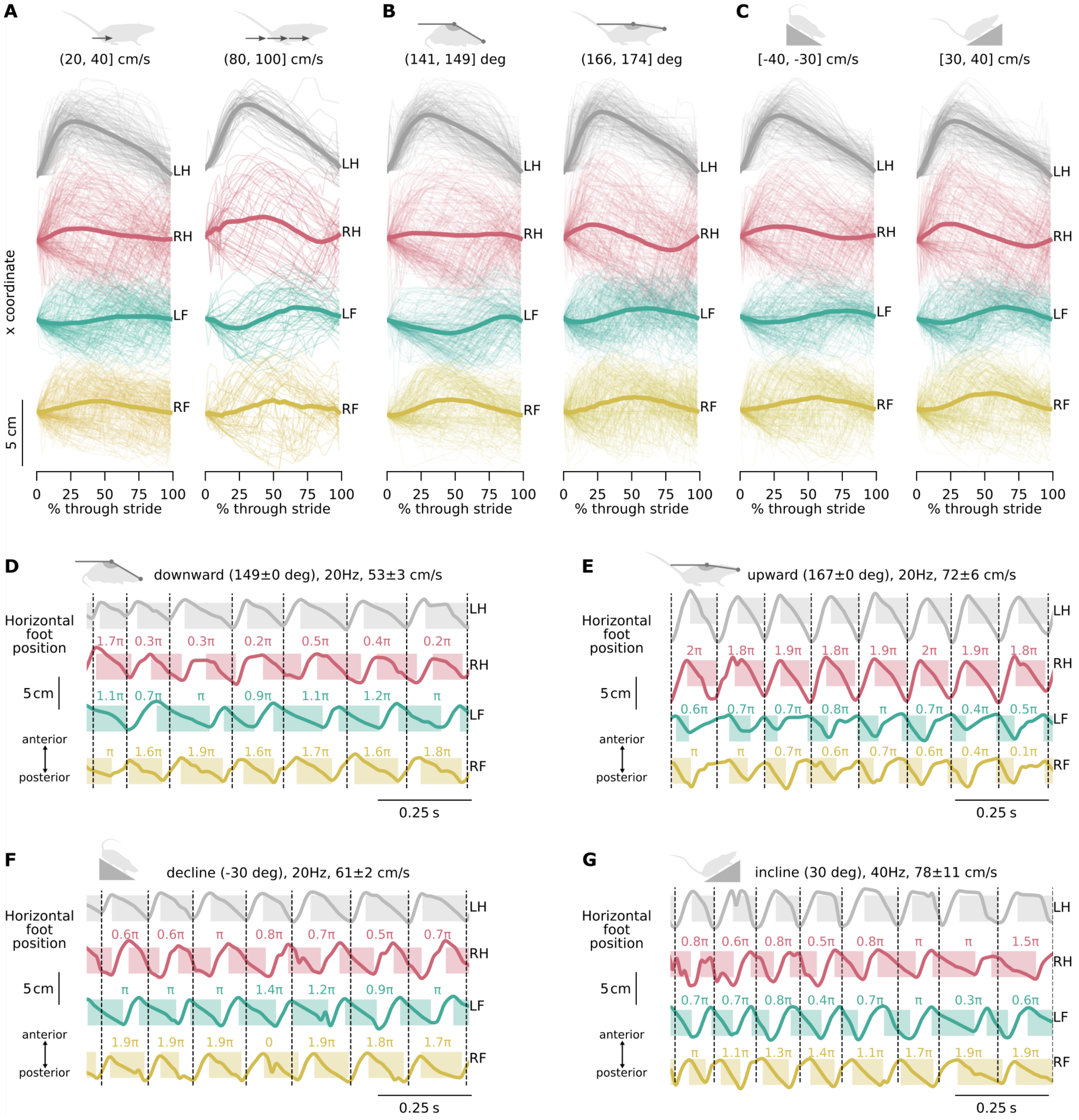

Figure S3: **Interlimb coordination is highly variable across strides, but distinct average patterns emerge across experimental conditions.** Related to Results section "Changes in posture and surface slope influence homolateral coordination, with limited effects of speed".

(A-C) Raw x-coordinate traces of all four limbs tracked by DeepLabCut, time-warped to the corresponding stride of the reference limb (here: left hindlimb, LH) at two ranges of speeds (A), snout-hump angles (B), and surfaces slopes (C). Shown are up to 200 randomly selected strides from a representative mouse (*thin lines*), as well as the average (*thick lines*) to illustrate stepping pattern heterogeneity. Limb phases were derived by autocorrelating within-stride traces of the reference limb against those of the other limbs. The scale from (A) applies to all panels.

(D-G) Example time series showing the horizontal coordinate of all four limbs during locomotion with a downward (D) and upward (E) oriented hump-snout vector in head height trials, and on a decline (F) and an incline (G) in slope trials. Speeds and snout-hump angles are averaged across the displayed strides and shown as mean±SEM. Vertical dashed lines partition the time series into strides based on LH swing onsets. Shaded areas denote stance phases, and limb phases relative to LH phase are annotated above the corresponding traces.

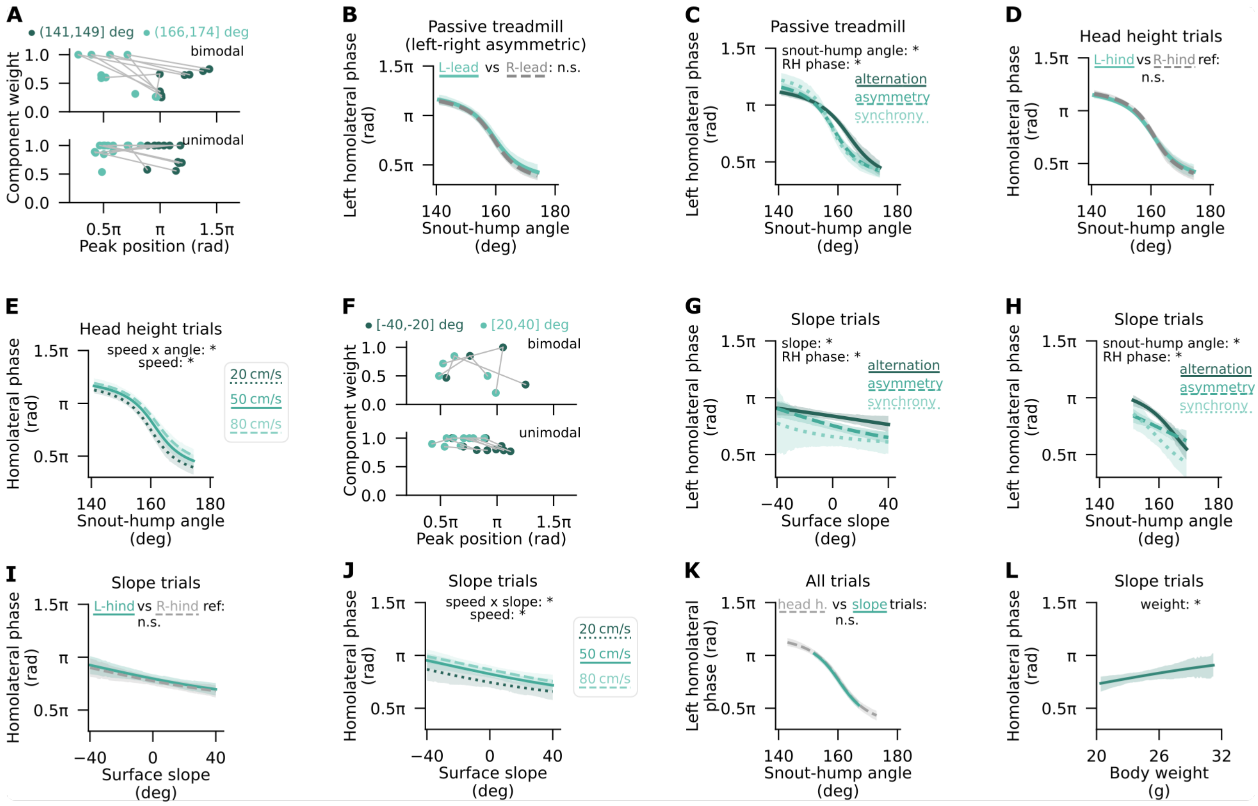

Figure S4: **Bilaterally symmetrical changes in homolateral phase align with modulation by anteroposterior leg load distribution.** Related to Results section "Changes in posture and surface slope influence homolateral coordination, with limited effects of speed".

(A, F) Component weights from optimal Von Mises mixture model fits to the homolateral phase data in two extreme snout-hump angle intervals (A) and surface slope intervals (F), plotted against the phase corresponding to the peak of the respective component's probability distribution. Points are colour-coded by snout-hump angle interval, with each point representing an individual mouse. Points for the same mouse are connected by grey lines. Note that peak position is a circular variable presented on a linear scale for convenience. Components with weights under 0.2 are considered negligible and not shown.

(B) Left homolateral phase as a function of snout-hump angle in strides characterised by asymmetric (left- or right-leading) hindlimb coordination. Shown are circular-linear mixed-effects regression fits to data from head height trials, including leading hindlimb as a predictor. Leading hindlimb HPD_{SSDO}=(-0.18,0.01).

(C, H) Left homolateral phase as a function of snout-hump angle in strides characterised by alternating, asymmetric (left- and right-leading combined), or synchronised hindlimb coordination. Shown are circular-linear mixed-effects regression fits to data from head height (C) and surface slope (H) trials, including hindlimb phase category as a predictor (head height trials: asymmetric-alternating HPD_{SSDO}=(0.20,0.36), synchronised-alternating HPD_{SSDO}=(0.09,0.36); slope trials: asymmetric-alternating HPD_{SSDO}=(0.24,0.38), synchronised-alternating HPD_{SSDO}=(0.35,0.73)). Left- and right-leading strides were pooled since the influence of snout-hump angle (panel B) and of slope (HPD_{SSDO}=(-0.43, 0.15)) on homolateral phase was statistically indistinguishable between lead-limb categories.

(D, I) Homolateral phase as a function of snout-hump angle (D) and surface slope (I) with both LH (solid teal traces) and right hindlimb (RH; dashed grey traces) used as the reference leg. Reference limb HPD_{SSDO} is (-0.07,0) and (-0.01,0.1) respectively. Shown are the fixed effects from circular-linear mixed-effects regression on data from surface slope trials. Shaded regions represent 95% highest posterior density intervals.

(E, J) Same as (D) and (I) but for three representative speeds. Speed HPD_{SSDO} is (-1.13,-0.95) and (-1.20,-1.00) respectively. Speed-angle interaction HPD_{SSDO}=(0.94,1.13), speed-slope interaction HPD_{SSDO}=(0.99,1.19).

(G) Same as (C) but as a function of surface slope. Hindlimb phase category HPD_{SSDO}=(0.91,1.56).

(K) Left homolateral phase as a function of snout-hump angle in head height and slope trials after accounting for trial type through circular-linear mixed-effects regression with trial type as a predictor (HPD_{SSDO}=(-0.03,0.08)).

(L) Same as (D) but as a function of animal body weight (HPD_{SSDO}=(-0.85,-0.63)).

Statistical significance criterion (Bayesian posterior interval): * HPD_{SSDO} interval does not include zero, n.s. otherwise.

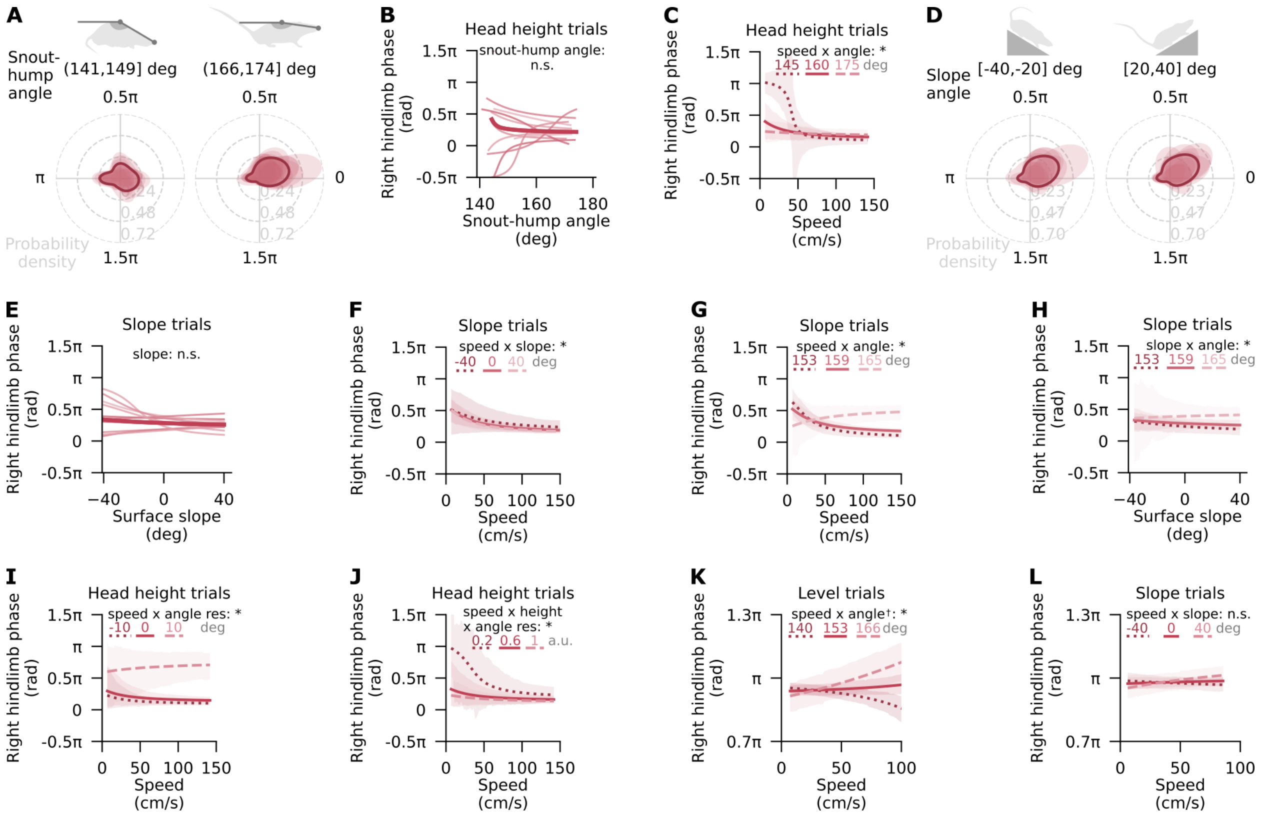

Figure S5: **Hindlimb coordination is influenced by total load and a posture-related variable in a speed-dependent manner.** Related to Results section "Changes in posture and surface slope influence homolateral coordination, with limited effects of speed" and Supplemental Information section "Supporting analysis of hindlimb coordination".

(A, D) Von Mises-smoothed distributions of right hindlimb (RH) phase relative to left hindlimb (LH) in two intervals of snout-hump angles (A) and surface slopes (D), shown for individual mice (shaded regions; n=10 mice) and as averages across mice (solid outlines). Limb phase values of 0 and π rad reflect limb synchrony and alternation respectively. Data from mice with fewer than 40 strides per category were excluded from the respective plot.

(B, E) RH phase as a function of snout-hump angle (B; HPD_{SSDO}=(-0.95,0.93)) and surface slope (E; HPD_{SSDO}=(-1.06,0.95)) with LH as the reference limb at median speed. Shown are circular-linear mixed-effects regression fits to individual mouse data (random effects; light traces) and the average (fixed effect; dark trace).

(C, F-G, I-J) RH phase as a function of speed at three snout-hump angles (C, G), surface slopes (F), residuals from snout-hump angle regression against the weight-adjusted head height (I), and weight-adjusted head heights (J) in head height (C, I-J) and slope (F, G) trials, with left hindlimb as the reference limb. Shown are circular-linear mixed-effects regression fits to single mouse data (random effects; light traces; n=12 mice) and the average (fixed effect; dark trace). All these variables showed significant interaction effects with speed (C: HPD_{SSDO}=(-0.99,-0.51), F: HPD_{SSDO}=(0.70,1.11), G: HPD_{SSDO}=(-1.11,-0.70), I: HPD_{SSDO}=(0.45,1.24), G: head height-speed interaction HPD_{SSDO}=(-1.18,1.23), head height-residuals-speed interaction HPD_{SSDO}=(0.45,1.24)).

(H) Same as (G) but as a function of surface slope. Interaction between slope and snout-hump angle HPD_{SSDO}=(-1.21,-0.64).

(K-L) RH phase as a function of speed at three representative snout-hump angles (K) and slopes (L) during level (K) and surface slope (L) trials on the motorised treadmill, with LH as the reference leg. Snout-hump angle interacted significantly with speed (K: HPD_{SSDO} = (-1.50,-1.24)), while slope did not (L: HPD_{SSDO} = (-1.97, 2.15)). Given the dominance of strict left-right alternation on the motorised treadmill, these results suggested that speed and posture modulate the extent of hindlimb phase lag, rather than the balance between hindlimb synchrony and alternation.

In all panels, phase data from left-injected mice were reflected across the origin to account for the stimulation-induced bias in left-right stepping order (see Figure S2 J-M). Data were also balanced for alternating (0.8-1.2 π rad) and advanced (0.2-0.8 π rad) left homolateral phases to minimise confounding between hindlimb coordination and the biomechanical variables of interest. † indicates that snout-hump angle is measured in non-restrained conditions and is not necessarily directly comparable to the snout-hump angle from head-fixed experiments

Statistical significance criterion (Bayesian posterior interval): * HPD_{SSDO} interval does not include zero, n.s. otherwise.

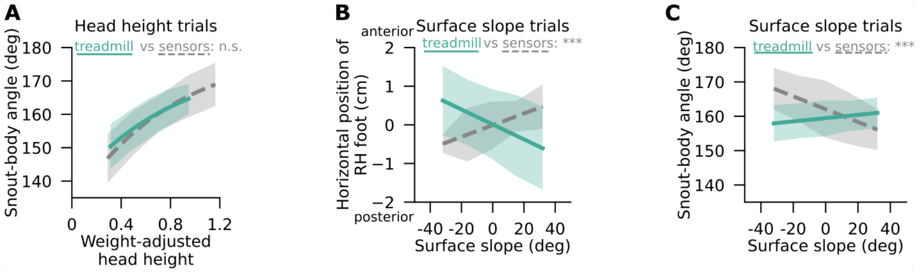

Figure S6: **Different postural adaptations to slope on force sensors and treadmill.** Related to Results section "Changes in posture and surface slope influence homolateral coordination, with limited effects of speed".
(A) Snout-hump angle as a function of weight-adjusted head height on the passive treadmill (solid teal line) and the force sensors (dashed grey line), approximated by exponential decay function. The shaded regions show 95% confidence intervals. For the passive treadmill, only data from stationary pre-stimulation periods are included. Statistical significance is assessed using a likelihood ratio test (p=0.15).
(B, C) Relative position of the right hindfoot (B; *Mann-Whitney U-test statistic U(7,12)=0,* $p=4\times10^{-5}$) or snout-hump angle (C; *Mann-Whitney U-test statistic U(7,12)=84,* $p=4\times10^{-5}$) as a function of surface slope on the passive treadmill (solid teal line) and the force sensors (dashed grey line), approximated by linear functions. The shaded regions show 95% confidence intervals.
Statistical significance refers to Mann-Whitney U-test or likelihood ratio test with thresholds: * $p < 0.05$, ** $p < 0.01$, *** $p < 0.001$.

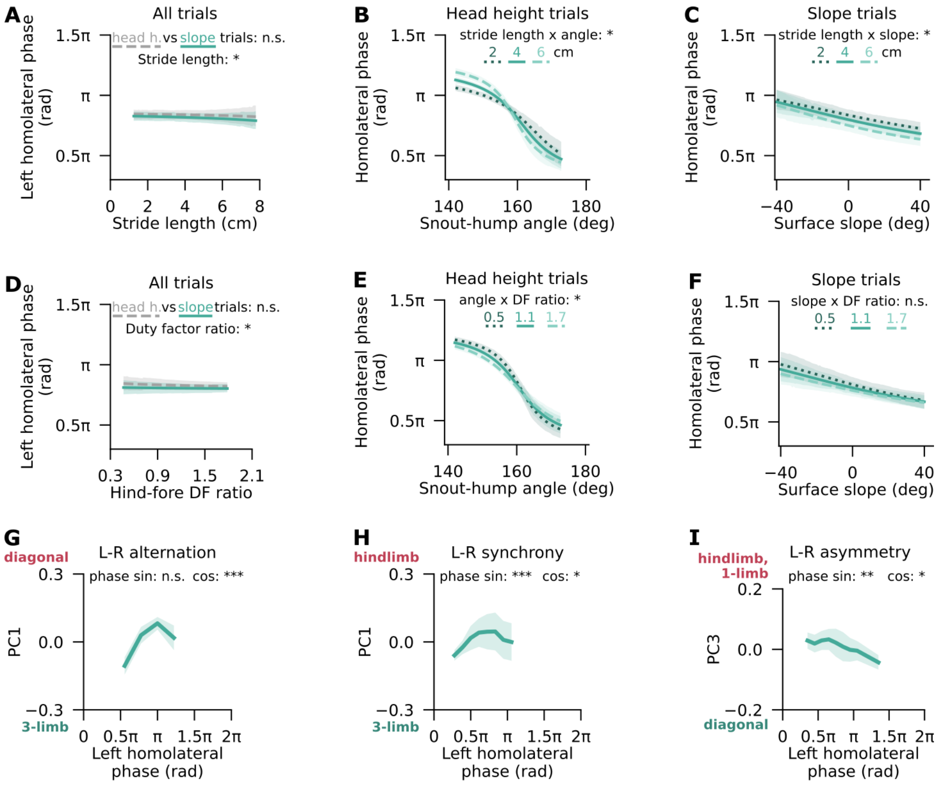

Figure S7: **Changes in homolateral phase are largely independent of speed and align with modulation by anteroposterior leg load distribution.** Related to Results section "Homolateral limb coordination is linked to changes in limb support patterns".
(A) Left homolateral phase as a function of stride length, with left hindlimb as the reference limb. Shown are the fixed effects from circular-linear mixed-effects regression on data from head height and slope trials, balanced for hindlimb phase and including trial type as a predictor. Shaded regions are 95% highest posterior density intervals. Stride length HPD_{SSDO}=(-1.2,-1.0).
(B, C) Homolateral phase as a function of snout-hump angle (B) and surface slope (C) at three stride lengths that span the range of observed values. Shown are the fixed effects from circular-linear mixed-effects regression analysis on data balanced for hindlimb phase. Shaded regions represent 95% highest posterior density intervals. Stride length interaction with snout-hump angle: HPD_{SSDO}=(1.0,1.2); with slope: HPD_{SSDO}=(1.0,1.2).
(D) Same as (A), but as a function of hind-fore duty factor ratio (HPD_{SSDO}=(1.4,1.6)).
(E, F) Same as (B, C), but for three representative hind-fore duty factor ratios. Duty factor ratio interaction with snout-hump angle: HPD_{SSDO}=(-1.1,-0.9); with slope: HPD_{SSDO}=(-1.2,1.1).
(G-I) Projections of limb support data onto the PCs identified in Figure 4 as significantly and consistently associated with snout-hump angle and surface slope, plotted as a function of left homolateral phase during alternating (G), synchronised (H), and asymmetric (I) hindlimb stepping on the passive treadmill. Shown are means across mice, with 95% confidence intervals, plotted only for phase values with above-average stride counts. The horizontal axis presents a circular variable on a linear scale for convenience.
Statistical significance criterion (Bayesian posterior interval): * HPD_{SSDO} interval does not include zero, n.s. otherwise.

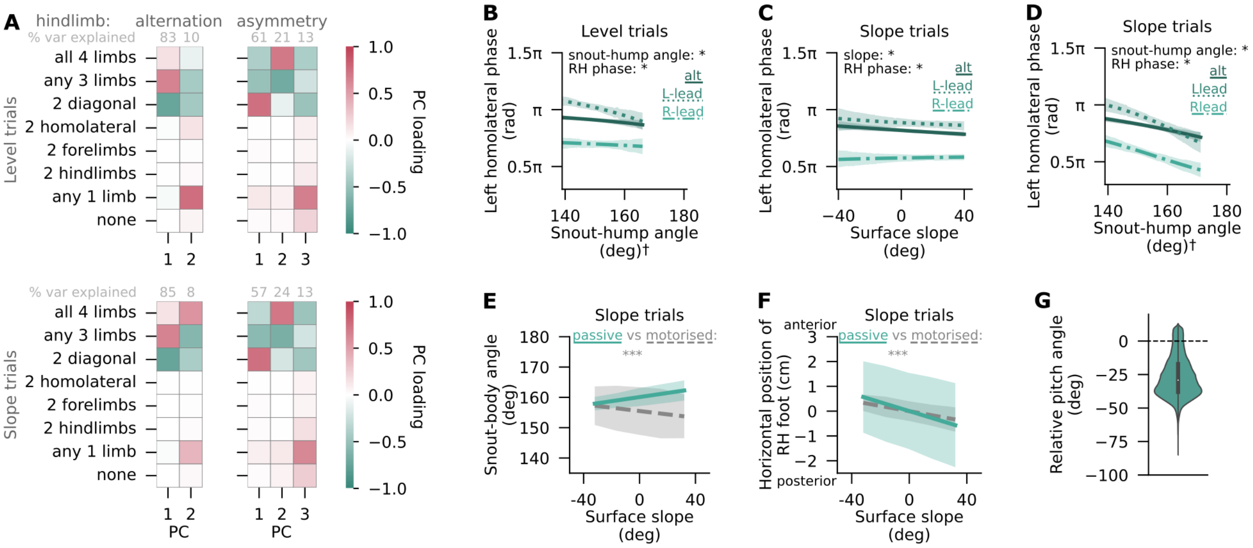

Figure S8: **Motorised and passive treadmill data differ partly due to distinct postural adaptations to slope, with no clear link to differences in left-right coordination.** Related to Results sections "Homolateral coordination during non-restrained locomotion is broadly consistent with modulation by load distribution" and "Homolateral limb coordination is linked to changes in limb support patterns".

(A) Loadings of the first 2-3 principal components collectively representing at least 90 % of the variance in limb support patterns during alternating (*left*) or asymmetric (*right*) hindlimb stepping in level (*top*) and surface slope (*bottom*) trials on the motorised treadmill. The variance explained by each component is shown above the heatmap.

(B-D) Left homolateral phase as a function of snout-hump angle (B,D) or surface slope (C) in strides characterised by alternating, left-leading, or right-leading hindlimb coordination on the motorised treadmill. Shown are circular-linear mixed-effects regression fits to data from level (B) and surface slope (C,D) trials, including hindlimb phase category as a predictor (level trials: left-leading-alternating HPD_{SSDO}=(-0.40,-0.28), right-leading-alternating HPD_{SSDO}=(0.57,0.70); slope trials: left-leading-alternating HPD_{SSDO}=(-0.33,-0.21), right-leading-alternating HPD_{SSDO}=(0.67,0.80)). Synchronous hindlimb coordination is not examined because it comprised fewer than 3% of all recorded strides.

(E-F) Snout-hump angle (E) and the relative position of the right hindfoot (F) as a function of surface slope during locomotion on the passive treadmill (*solid teal line*) and motorised treadmill (*dashed grey line*), approximated by linear regression. The shaded regions show 95% confidence intervals. Statistical significance is assessed using a likelihood ratio test.

(G) Distribution of head pitch angles recorded during non-restrained locomotion relative to the head tilt during head fixation.

† indicates that snout-hump angle is measured in non-restrained conditions and is not necessarily directly comparable to the snout-hump angle from head-fixed experiments.

Statistical significance criterion (Bayesian posterior interval): * HPD_{SSDO} interval does not include zero, n.s. otherwise.
